# Evidence for a Non-Catalytic Ion-Binding Site in Multiple RNA-Dependent RNA Polymerases

**DOI:** 10.1371/journal.pone.0040581

**Published:** 2012-07-11

**Authors:** Heli A. M. Mönttinen, Janne J. Ravantti, Minna M. Poranen

**Affiliations:** 1 Department of Biosciences, University of Helsinki, Helsinki, Finland; 2 Institute of Biotechnology, University of Helsinki, Helsinki, Finland; University of Edinburgh, United Kingdom

## Abstract

A high-affinity divalent cation-binding site located proximal to the catalytic center has been identified in several RNA-dependent RNA polymerases (RdRps), but the characteristics of such a site have not been systematically studied. Here, all available polymerase structures that follow the hand-like structural motif were screened for the presence of a divalent cation close to the catalytic site but distinct from catalytic metal ions. Such non-catalytic ions were found in all RNA virus families for which there were high-resolution RdRp structures available. Bound ions were always located in structurally similar locations at an approximate 6-Å distance from the catalytic site. Furthermore, the second aspartate residue in the highly conserved GDD sequence was found to be involved in the coordination of the bound ion in all viral RdRps studied. These results suggest that a non-catalytic ion-binding site is conserved across positive-sense, single-stranded, and double-stranded RNA viruses. Interestingly, a non-catalytic ion was also observed in a similar position in the reverse transcriptase of the human immunodeficiency virus. Moreover, two members of the DNA-dependent DNA polymerase B family displayed an ion at a comparable distance from the catalytic site, but the position was clearly distinct from the non-catalytic ion-binding sites of RdRps.

## Introduction

Viral RNA-dependent RNA polymerases (vRdRps) are essential for the replication of RNA genomes and the transcription of viral messenger RNAs (mRNAs). Like DNA-dependent DNA polymerases (DdDps) and the reverse transcriptases (RTs) of retroviruses, the vRdRps of double-stranded RNA (dsRNA) and single-stranded RNA (ssRNA) viruses with positive-sense genomes follow the canonical right-handed structure with fingers, palm, and thumb domains [1]–[7]. The structural conservation and sequence level identities indicate that the right-handed polymerases share a common origin [7].

**Figure 1:**
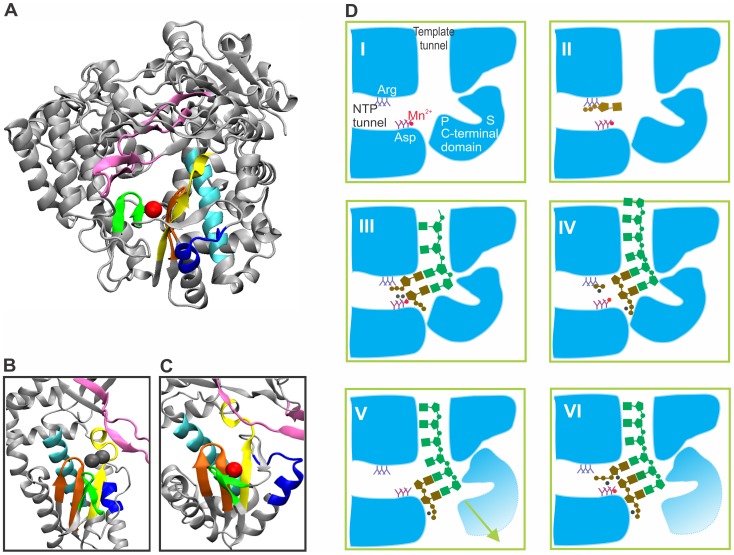
An overview of the vRdRp structure, the catalytic site, and the role of the non-catalytic ion. (A) Conserved motifs in the phage φ6 RdRp (PDBid: 1HHS) and magnified views of the catalytic sites from the RdRps of hepatitis C virus (B) and phage φ6 (C) (PDBids: 2WHO and 1HHS, respectively). Yellow, motif A; cyan, motif B; orange, motif C; blue, motif D; green, motif E; and mauve, motif F. A bound non-catalytic ion is shown as a red sphere (A and C), and the catalytic ions are depicted as gray spheres (B). The structural models were visualized using program VMD 1.8.7 [30]. A schematic model for the initial steps of φ6 RdRp-catalyzed RNA polymerization (D). (I) A cartoon representation of a cross-section of the φ6 RdRp with a bound non-catalytic Mn^2+^ ion (PDBid: 1HHS). The template pocket and initiation platform are designated as S and P, respectively. A Mn^2+^ ion is presented as a red sphere, and the conserved aspartate residues of the catalytic site are depicted. (II) A preinitiation complex (PDBid: 1HI1). An entering NTP (brown) bound to the arginine/lysine-rich interrogation site. (III) Assembly of the initiation complex (PDBid:1HI0). The catalytic Mg^2+^ ions (gray spheres) are located proximal to the catalytic site. The entry of the template (green) and the correct coordination of NTP for catalysis are dependent on the non-catalytic Mn^2+^ ion. (IV) Catalysis. A phosphodiester bond is formed between the first two nucleotides of the daughter strand (brown). Pyrophosphate together with bound Mg^2+^ diffuse from the catalytic site (PDBid:1UVK). The second catalytic Mg^2+^ remains bound to the 5′-triphosphate group of the daughter strand. (V) Structural changes in the C-terminal domain allow the exit of the dsRNA product from the catalytic site. The non-catalytic ion is lost from its position (PDBid: 4A8S). (VI) Subsequent catalytic steps in elongation. The model is based on data presented in references [6], [16], [17], [56].

Six conserved structural motifs (A–F) have been identified in the vRdRps of dsRNA and positive-sense ssRNA viruses [7]–[10]. Four of these motifs (A–D) are common to all hand-shaped polymerases [7], [10] and are located in the palm domain, which consists of four antiparallel β-strands and two α-helices. One of these β-strands overlaps with motif A and two β-strands overlap with motif C [7] (Fig. 1A). Both motifs A and C contain the strictly conserved catalytic aspartates that are needed for the coordination of the catalytic Mg^2+^ ions [11], [12]. The catalytic aspartate of motif C is the first aspartate of the GDD sequence that is characteristic of both vRdRps and RTs with some modifications in the first position (e.g., the MDD sequence in the human immunodeficiency virus and the SDD sequence in the pseudomonas phage φ6) [10]. Birnaviruses (a family of dsRNA viruses) are exceptional among RNA viruses, as their RdRps have an ADN sequence at an equivalent structural position [9], [13], and the motif order A-B-C has been permutated to the order C-A-B [14]. Motifs E and F, which have been identified in vRdRps and RTs, are located at the boundary of the palm and thumb motifs and in the finger domain, respectively [8], [15] (Fig. 1A).

The catalytic mechanism is strikingly similar in all nucleic acid polymerases despite their structural differences and substrate specificities. All known polymerases are dependent on two Mg^2+^ ions, assigned as catalytic ions A and B [12] (see Fig. 1B). Catalytic ion A is responsible for the activation of nucleotides in the nucleophilic reaction, whereas ion B directs the departure of the pyrophosphate moiety (for details, see [12]). In addition, a third divalent cation (Mn^2+^), which is located approximately 6 Å from the catalytic site in close proximity to the β-sheets in the A and C motifs (Fig. 1A and 1C), was identified in the initial structural analysis of the pseudomonas phage φ6 RdRp and designated as a non-catalytic ion [6]. The presence of this ion (as well as catalytic ions A and B) appears to be dependent on the conditions applied during such analyses [6], [16], [17] (compare structures in Fig. 1B and 1C). Subsequent structural studies of other vRdRps revealed bound ions at positions similar to the non-catalytic ion site in pseudomonas phage φ6 (see e.g. [18], [19]), but the conservation of the site occupied by the metal ligand has not been systematically analyzed.

Previous biochemical and structural studies have shown that the bound non-catalytic ion has an essential role in RNA polymerization (see Fig. 1D) [16], [17]. A mutation in one of the amino acids that coordinates the bound Mn^2+^ in pseudomonas phage φ6 RdRp results in decreased Mn^2+^-binding affinity, increased Mn^2+^ dependence of RNA polymerization, reduced structural flexibility, and lower template-RNA binding. Replacement of Mn^2+^ by Mg^2+^ induces structural stabilization (increased thermal stability), preventing template entry [17]. Most importantly, the establishment of the correct geometry of nucleotide triphosphates (NTPs) for catalysis is abolished in the absence of the non-catalytic ion [16]. Furthermore, our recent structural and biochemical studies proposed that the Mn^2+^ dependence of φ6 RdRp-catalyzed RNA polymerization arises from the transient loss of the bound non-catalytic ion and the requirement for ion rebinding [17] (Fig. 1D). Interestingly, the activities of other vRdRps are also significantly stimulated by Mn^2+^ ions (see references [20]–[28]), but the underlying mechanism behind this phenomenon is not yet understood.

In this study, we address the conservation of the non-catalytic ion-binding site in vRdRps and present simple geometrical constraints for the identification of non-catalytic ion-containing RdRp structures. Initially, all existing vRdRp structures were screened for an ion located in the position corresponding to the non-catalytic ion-binding site of pseudomonas phage φ6. We identified 32 vRdRp structures having such an ion. Those structures represent 10 viral species from seven viral families. To study the conservation of the coordination pattern for the bound ion, we combined the coordination data of the ion with the structural alignment information of vRdRps. This analysis revealed that the highly conserved second aspartate of the GDD sequence is required for the coordination of the non-catalytic ion. In addition, we screened all hand-shaped RT, DdDp, and DNA-dependent RNA polymerase (DdRp) structures having ions close to the catalytic site. This screen revealed a human immunodeficiency virus (HIV) RT structure with an ion bound to the position comparable to the non-catalytic ion-binding site in vRdRps. In addition, DdDps of DNA polymerase family B from escherichia phage RB69 and *Saccharomyces cerevisiae* contained ions at an approximate 6-Å distance from the catalytic site, but the position was distinct from the position of the non-catalytic ions in vRdRps. Our results suggest that the second aspartate of the GDD sequence that is present in vRdRps and RTs but not in DNA-directed polymerases is the fingerprint for the non-catalytic ion-binding site in RNA-directed polymerases. Furthermore, based on existing structural data, the type of bound non-catalytic ion and its presence in solved X-ray structures are highly dependent on the applied crystallization conditions.

## Methods

### Screening of Polymerase Structures for the Presence of Bound Non-catalytic and Catalytic Ions

The vRdRp, RT, and hand-shaped DdDp and DdRp structures obtained by X-ray crystallography were collected from the Protein Data Bank (PDB) (http://www.pdb.org; structures before 19.4.2011), and structures containing cations were selected using suitable PDB Search options. First, the RT, DdDp, and DdRp structures were screened by visual inspection for the presence of cations in the palm domain. The collected cation-containing polymerase structures (see Table S1) were aligned structurally using the program Multiseq [29]; the bound cation ligands were marked to reveal i) the presence of a cation bound within the palm domain in the proximity of the conserved adjacent β-sheets in the A and C motifs to identify putative non-catalytic ions (Fig. 1C) and ii) the presence of catalytic ions proximal to the loop regions in the A and C motifs (Fig. 1B). The pseudomonas phage φ6 RdRp structure (PDBid: 1HHS) was used as a reference for the position of the non-catalytic ion (Fig. 1C), and the RdRp structure of the hepatitis C virus (PDBid: 2WHO) was used as a reference for the positions of the catalytic ions (Fig. 1B). The RdRp structures were also analyzed for bound NTPs, nucleic acid molecules, and drug compounds. Furthermore, information on the purification and crystallization conditions was gathered from the primary references for each structure.

### Identification of the Non-catalytic Ion-binding Site

The positional conservation of the non-catalytic ion-coordinating amino acids was determined by the structural alignment of the selected vRdRp and RT structures using the program Multiseq [29]. The information on the amino acids involved in the coordination of the bound metal ion was obtained from the corresponding PDB files.

### Measurements of the Distances between the Non-catalytic and Catalytic Ions

The distances between the non-catalytic and catalytic ions were determined by structurally aligning each non-catalytic ion containing vRdRp with the RdRp of the hepatitis C virus (PDBid: 2WHO) containing the catalytic ions (Fig. 1B). The hepatitis C virus RdRp structure was selected as a reference, because it was previously used for the determination of the catalytic site of pseudomonas phage φ6 RdRp [6]. The distances between the third ion and the catalytic ions in DdDps were measured from ions present in the same structure. The distances were determined using distance tools from the program VMD 1.8.7 [30].

### Determination of the Distance Parameters for the Identification of the Non-catalytic Ions

A locally written Python-script was used to optimize the distance parameters to allow the geometrical identification of structures containing non-catalytic ions and the rejection of structures containing ions in other structural positions only. The script enables i) the definition of spheres that have a structurally defined center (measuring point) and radius, ii) the selection or rejection of structures that contain ions within the intersection of two or more spheres, and iii) the combination of different selection or rejection criteria. The number of spheres, the positions of the measuring points, and the length of the radius for each sphere were optimized using the set of vRdRp structures presented in Table 1 (57 structures with either catalytic or non-catalytic ions, or both) until the strictest parameters that were able to provide all the non-catalytic ion-containing structures without false positives could be identified. The optimized parameters were also tested for RT structures.

**Table 1:** Number of vRdRp structures with ions or other elements proximal to the catalytic site.

Elements other than ions^1^	Structures withno ions	All structures witha non-catalytic ion	All structures withcatalytic ion(s)^3^	Non-catalytic and catalyticion(s)^3^ present in the samestructure^5^
No other elements (64)	47	14	2, (2)	1
NTP(s)^2^ (15)	8	–	7	–
Template and NTP(s)^2^ (16)	3	7	9, (2)	4, (1)
Template (23)	9	11	3, (2)	(2)
Drug compound^4^ (75)	69	–	5, (1)	–
Total number of vRdRp structures (193)	136	32	26, (7)	5, (3)

1 The total number of structures is given in parenthesis.

2 Modified and unmodified NTPs.

3 Number of structures in which only one catalytic ion is present is in parentheses.

4 Other than nucleotide derivatives.

5 These structures are also included in the two previous columns.

### Sequence Alignment of Polymerases

Multiple sequence alignments were carried out for non-putative vRdRp amino acid sequences from the different members of the *Reoviridae, Cystoviridae*, and *Leviviridae* families. The members of the *Sedoreovirinae* and *Spinareovirinae* subfamilies of the *Reoviridae* family were analyzed separately. Amino acid sequences for multiple sequence alignments were selected from the National Center for Biotechnology Information (NCBI) protein database (http://www.ncbi.nlm.nih.gov/protein/). As the *Cystoviridae* family has only one genus (*Cystovirus*), all the polymerase sequences annotated for cystoviruses were included in the analysis (5.7.2011) (see Table S2). For alignments of vRdRp sequences representing members of the *Sedoreovirinae* and *Spinareovirinae* subfamilies and the *Leviviridae* family (5.7.2011), only sequences from species assigned by the International Committee on Taxonomy of Viruses (ICTV) (http://talk.ictvonline.org) were selected (see Table S2). One vRdRp sequence from each species of the *Leviviridae* family and one vRdRp sequence from each genus in the *Sedoreovirinae* and *Spinareovirinae* subfamilies were chosen (see Table S2). Sequences were aligned using the MUSCLE-Multiple alignment tool [31].

## Results

In the following sections, we describe analyses that were based on published X-ray structures on hand-shaped polymerases and their annotations available in the PDB. Although it is possible that the PDB entries contain misannotations, we are confident that sporadic errors in one or a few of the structures do not have a significant effect on the overall results presented on the non-catalytic ions in vRdRps.

### High-resolution Viral RdRp Structures

Currently, there are 193 high-resolution vRdRp structures available from 16 different viral species belonging to seven viral families (Table 2 and Table S1). These structures represent 159 vRdRps of positive-sense ssRNA viruses and 34 vRdRps of dsRNA viruses. However, there are no high-resolution structures of vRdRps of negative-sense RNA viruses. For the hepatitis C virus, there are 94 different RdRp structures denoted in the PDB, whereas some viruses are represented by only two RdRp structures (i.e., rabbit hemorrhagic virus and Dengue virus). Several of the available vRdRp structures had a bound drug compound (75 structures), template nucleic acid (23 structures), one or two NTPs (15 structures), or both template and NTPs (16 structures), whereas others had none of these ligands (Table 1 and Table S1). Approximately one-third of the RdRp structures (75 out of 193) contained one or more cations (Table 1); in 58 structures, the observed ion was located proximal to the catalytic site. In the remaining structures, cations were observed in the finger subdomain (RdRps of poliovirus, Dengue virus, West Nile virus, and rhinovirus), thumb subdomain (RdRps of the Dengue virus and West Nile virus), and the surface of the structure (RdRps of the hepatitis virus).

**Table 2:** A list of virus species having RdRp structures in the PDB, observed non-catalytic ion types, and distances of non-catalytic ions from the catalytic site.

Family	Genus	Species^1,2^	Observed non-catalytic iontypes(PDBid)^3,4^	Distance (Å) of non-catalytic ions from the catalytic position^5^
*Caliciviridae*	*Lagovirus*	**RHV**	Lu^2+^(**1KHV**)	6.0
	*Norovirus*	**Norwalk virus**	Mg^2+^(**1SH3**), Mn^2+^ (3H5Y, 3H5X)	6.6
	*Sapovirus*	Sapporo virus	–	–
*Flaviviridae*	*Flavivirus*	**Dengue virus**	Mg^2+^(**2J7U**)	5.8
		**West Nile virus**	Ca^2+^(**2HCN**), Mg^2+^ (2HFZ)	5.7
	*Hepacivirus*	hepatitis C virus	–	–
	*Pestivirus*	BVDV	–	–
*Leviviridae*	*Allolevivirus*	**Qβ**	Ca^2+^ (**3AGP**), Mg^2+^ (3AGQ)	4.0
*Picornaviridae*	*Aphthovirus*	**FMDV**	Mg^2+^(**1WNE**, 2EC0, 2E9Z, 2E9T,2E9R, 3KOA, 3KNA, 3KLV, 3KMS)	6.1
	*Enterovirus*	coxsackie virus	–	–
		**poliovirus**	Ca^2+^(**1RDR**)	4.9
		human rhinovirus	–	–
*Birnaviridae*	*Avibirnavirus*	**IBDV**	Mg^2+^(2R72)	5.5
*Cystoviridae*	*Cystovirus*	**φ6**	Mg^2+^(1HI8), Mn^2+^ (1HI0, **1HHS**,1HHT, 1UVI, 1UVJ, 1UVK, 1UVL,1UVN, 1UVM, 2JLF)	6.4
*Reoviridae*	*Orthoreovirus*	**reovirus**	Mn^2+^(**1MWH)**	6.1
	*Rotavirus*	simian rotavirus	–	–

1 Abbreviations used: RHV, rabbit hemorrhagic virus; BVDV, bovine viral diarrhea virus; Qβ, enterobacteria virus Qβ; FMDV, foot-and-mouth disease virus; coxsackie virus, human enterovirus B; IBDV, infectious bursal disease virus; φ6, pseudomonas phage φ6; reovirus, mammalian orthoreovirus.

2 The viral species for which a non-catalytic metal was found are in bold.

3 PDBids are given only for structures that include a non-catalytic ion.

4 Structures used for the determination of the distances are in bold.

5 Structures were structurally aligned with the hepatitis C virus RdRp structure (PDBid: 2WHO). Distances were measured from catalytic ion A of the hepatitis C virus RdRp.

### Non-catalytic Ion-binding Sites in Viral RdRps

**Figure 2:**
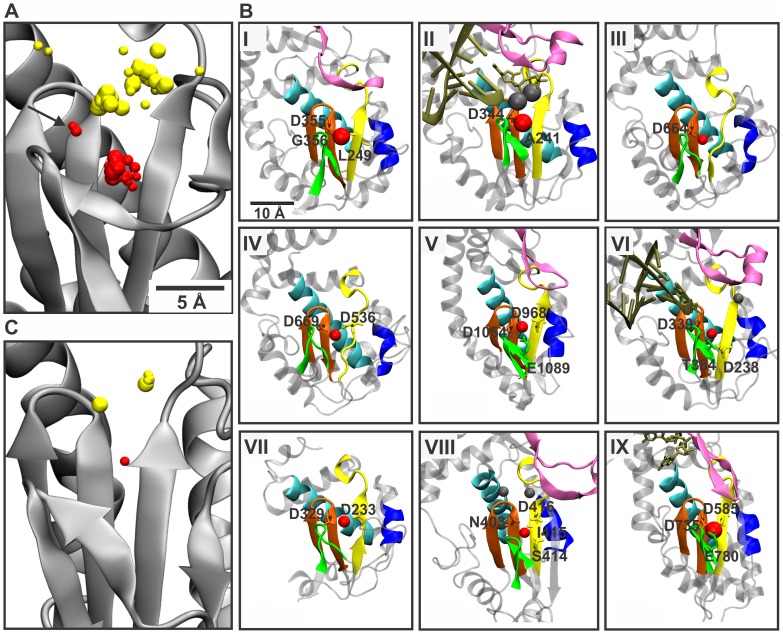
Non-catalytic and catalytic ions in vRdRps. (A) Structural alignment of vRdRps with bound cations. The catalytic site of the φ6 RdRp (PDBid: 1HHS) polypeptide is depicted with cations from all vRdRp structures. The catalytic ions are colored yellow, and the proposed non-catalytic ions are depicted as red (A and C). The size of the spheres depends on the type of the metal ion ligand. The arrow indicates the position of the non-catalytic ion in the enterobacteria phage Qβ RdRp structures. (B) Magnified views of the catalytic site from selected viral RdRp structures with bound divalent cations. The vRdRp motifs are colored as in Figure 1. Template nucleic acids and nucleotides are shown in tan. Non-catalytic ions are shown as red spheres, and catalytic ions are shown as gray spheres. The amino acids involved in the coordination of the bound non-catalytic ion are indicated. RdRp of (I) rabbit hemorrhagic virus with a Lu^2+^ ion (PDBid: 1KHV); (II) Norwalk virus with three Mn^2+^ ions (PDBid: 3H5Y); (III) Dengue virus with a Mg^2+^ ion (PDBid: 2J7U); (IV) West Nile virus with a Ca^2+^ ion (PDBid: 2HCN); (V) enterobacteria phage Qβ with a Ca^2+^ ion (PDBid: 3AGP); (VI) foot-and-mouth disease virus with three Mg^2+^ ions (PDBid: 2E9T); (VII) poliovirus with a Ca^2+^ ion (PDBid: 1RDR); (VIII) infectious bursal disease virus with three Mg^2+^ ions (PDBid: 2R72); and (IX) mammalian orthoreovirus 3 with a Mn^2+^ ion (PDBid: 1MWH). (C) The catalytic site of the HIV RT structure 1N6Q with cations from all HIV RT structures. The molecules were visualized using program VMD 1.8.7 [30].

All vRdRp structures containing cations in the palm subdomain were structurally aligned to compare the positional distribution of the bound ligands. Two distinct ion clusters were observed (Fig. 2A): one was between the β-strands of motifs A and C, and the other was in the catalytic site (see the reference structures in Fig. 1). The ions of the first cluster were all located within a few Ångströms of the position corresponding to the non-catalytic ion-binding site of pseudomonas phage φ6 RdRp (Fig. 1A and 1C). This suggests that a metal ligand-binding site that is distinct from the positions of the catalytic ions is common among vRdRps from different species. Such locations were defined as non-catalytic ion-binding sites, and the bound ion was termed a non-catalytic ion. A representative structure from each viral family depicting the position of the metal ion ligand is shown in Figure 2B (see Fig. 1C for the position of the non-catalytic ion in pseudomonas phage φ6 RdRp, representing family *Cystoviridae*).

Visible outside of the tight cluster of the non-catalytic ions were two single cations from enterobacteria virus Qβ RdRp structures 3AGQ and 3AGP (Fig. 2A). Nevertheless, the position of the ion between the β-strands of motifs A and C (Fig. 2B, panel V) was highly similar to the non-catalytic ion-binding site in other vRdRps (Fig. 2B). We thus propose that the observed ion in these Qβ RdRp structures is a non-catalytic ion as was suggested in the original description of these structures [32].

This structural alignment allowed us to identify 32 vRdRp structures with bound non-catalytic ions (Fig. 2A and 2B). These RdRps represented 10 viral species from seven viral families (Table 2 and Table S1). Thus, at least one RdRp structure from each viral family included in our analysis displayed a non-catalytic ion. Within a single family (e.g., *Picornaviridae*), there were members that had an ion bound to the position equivalent to the non-catalytic ion site in pseudomonas phage φ6 RdRp (poliovirus and foot-and-mouth disease virus) and those in which no ion was detected (human rhinovirus and coxsackie virus; Table 2). Moreover, ion-bound and non-bound structures were described for RdRps representing a single virus species (e.g., pseudomonas phage φ6, poliovirus, and foot-and-mouth disease virus; see Table S1).

Of the 32 identified vRdRp structures with bound divalent metal ions in the non-catalytic ion-binding site, 14 contained neither template nor NTPs (RdRps of Dengue virus, West Nile virus, enterobacteria phage Qβ, poliovirus, infectious bursal disease virus, and pseudomonas phage φ6); 18 were binary complexes with bound template or template plus NTP. One of these was an initiation complex having a template and two NTPs bound proximal to the catalytic site. Despite the large number of RdRp structures containing drug compounds, none of these complexes had metal ions bound to the non-catalytic ion-binding site (Table 1). Taking into account all available vRdRp structures (Table 1 and Table S1), the presence of bound non-catalytic ions did not appear to correlate with any specific stage of polymerization.

### Catalytic Ions in vRdRps

Catalytic ions were not significantly observed more often than non-catalytic ions (Table 1). The total number of vRdRp structures having catalytic ions was 33, seven of which contained only one catalytic ion (Table 1). The cluster of catalytic ions was more diffuse that the main cluster of non-catalytic ions (Fig. 2A). In several RdRp structures, the observed catalytic ions were clearly shifted from the locations that they occupied in the RdRp structure of the hepatitis C virus (PDBid: 2WHO) (which, in this study, was used as a reference for defining the catalytic position; Fig. 1B) towards the thumb subdomain (e.g., compare Fig. 2B, panels VIIII and II). Although diffuse, the cluster of ions at the catalytic site was clearly bipartite, reflecting the distinct positions of catalytic ions A and B.

### Distances between the Catalytic and Non-catalytic Ions

To obtain comparable distance measurements for the observed non-catalytic ions from the catalytic site, the representative vRdRp structures from all the viral species identified in Table 2 were superimposed on the structure of the hepatitis C virus RdRp containing catalytic ions (PDBid: 2WHO). Comparison of the metal ion-binding site within a viral species was not feasible due to the low occupancy of catalytic ions (see above; Table 1 and Table S1). On average, catalytic ions A and B were 5.5 Å and 7.3 Å away from the position of the non-catalytic ion, respectively (see Table 2 for the variability in distance from catalytic ion A).

### The Coordinating Amino Acids

**Figure 3:**
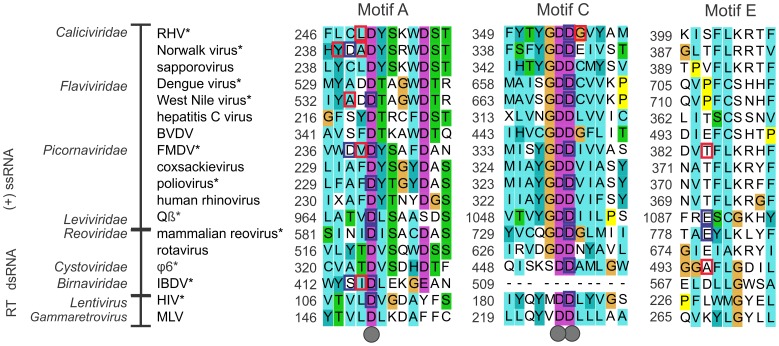
The amino acid sequences of motifs A, C, and E from the structural alignment. The number of the first residue in each motif is given. The amino acids that coordinate the catalytic ion are indicated with grey dots (below the alignment). The residues that coordinate the non-catalytic ion in different polymerases are marked with squares. Blue squares indicate that the amino acid coordinates the non-catalytic ion with its side chain, and red squares indicate that the main chain coordinates the ion. The names of the viral species and families are shown on the left. Abbreviations used: RHV, rabbit hemorrhagic virus; BVDV, bovine viral diarrhea virus; FMDV, foot-and-mouth disease virus; coxsackie virus, human enterovirus B; Qβ, enterobacteria virus Qβ; φ6, pseudomonas phage φ6; IBDV, infectious bursal disease virus; HIV, human immunodeficiency virus; and MLV, murine leukemia virus. The non-catalytic ion is observed in species marked with (*). The alignment is colored according to ClustalX 2.0.12 default settings [57].

According to the PDB entries, the number of amino acids that were identified to be involved in the coordination of non-catalytic ions in different vRdRps varied from one to four. To study the conservation of these amino acid residues, we structurally aligned all vRdRps for which there was high-resolution structural information available (a representative structure was selected for each vRdRp). Alignment information was subsequently combined with the existing metal coordination information from the PDB. Three structural positions were identified to be involved in the coordination of the bound non-catalytic ion in the majority of the vRdRp structures: one located in motif A, the second in motif C, and the third in motif E (Fig. 3).

The position of the coordinating amino acid varied slightly in motif A within –3 residues from the site of the invariant aspartate involved in the coordination of the catalytic ions (Fig. 3). Interestingly, this conserved aspartate also interacted with the non-catalytic ion in six vRdRps and a closely positioned aspartate residue in two vRdRps. However, in most other vRdRps, the non-catalytic ion was coordinated by a backbone carbonyl oxygen within motif A, suggesting that the position, rather than the type of amino acid, is critical for the establishment of the metal ion-binding site.

The amino acid proximal to the catalytic aspartate of motif C participates in the coordination of the non-catalytic ions in all vRdRps with bound ions, irrespective of their origins (Fig. 3). This position corresponds to the second aspartate residue in the highly invariant GDD sequence, which is typical for vRdRps. The infectious bursal disease virus RdRp, containing an asparagine residue in the ADN sequence that structurally corresponds to the GDD sequence of other vRdRps, also coordinates a bound non-catalytic ion (Fig. 2B, panel VIII). Based on the information presented in the PDB, the same aspartate residue also interacts with catalytic ion A if located in its canonical position (see Table S1). In the Norwalk virus RdRp structure (PDBid: 3H5Y; Fig. 2B, panel II) in which both catalytic ion A and the non-catalytic ion are present, the second aspartate residue of the GDD sequence interacts with both ions. Consequently, this aspartate residue appears to form a direct link between the two divalent cations.

In addition to the conserved aspartates of motifs A and C, a glutamic acid in motif E coordinates the non-catalytic ion in the RdRps of the mammalian reovirus and bacteriophage Qβ [32], [33]. Rotavirus, which belongs to the same viral family as reovirus, also has a glutamic acid in a similar position (Fig. 3). To study the conservation of this site in other viruses related to either reovirus or bacteriophage Qβ, we carried out multiple sequence analyses for selected members of the *Reoviridae* and *Leviviridae* families. We found that the glutamic acid of motif E is conserved in ortoreovirus, rotavirus, and orbivirus genera of the *Reoviridae* family as well as in allolevivirus and levivirus genera of the *Leviviridae* family (data not shown). The backbone carbonyl oxygen, at an equivalent structural position as in motif E, interacts with the bound non-catalytic ion in foot-and-mouth disease virus and pseudomonas phage φ6 RdRps (Fig. 3). Furthermore, the RdRp of bacteriophage φ6 has a non-catalytic ion coordinated by a glutamic acid proximal to motif E [6], which appears to be conserved in all known cystoviruses except in bacteriophage φ8, which has an aspartate at an equivalent position (data not shown).

### Non-catalytic Ions in other Hand-shaped Polymerases

In the PDB, there are only six RT structures with divalent cations proximal to the catalytic site. Structural alignment of these RTs (Fig. 2C) revealed similar positional patterns of ions as observed in vRdRps (Fig. 2A) and allowed the identification of an HIV RT structure (PDBid: 1N6Q) with a Mg^2+^ ion bound in a position similar to the non-catalytic ion-binding sites of vRdRps. The distance between the non-catalytic and catalytic ions present in another HIV RT structure (PDBid: 2IAJ) was 5.8 Å (Fig. 2C), matching the distances observed in vRdRps (Table 2). In addition, the coordination pattern of this ion was strikingly similar to the non-catalytic ions of RdRps. The first conserved aspartate of motif A and the second conserved aspartate of motif C interact with the bound ion (Fig. 3; see Table S3).

**Figure 4:**
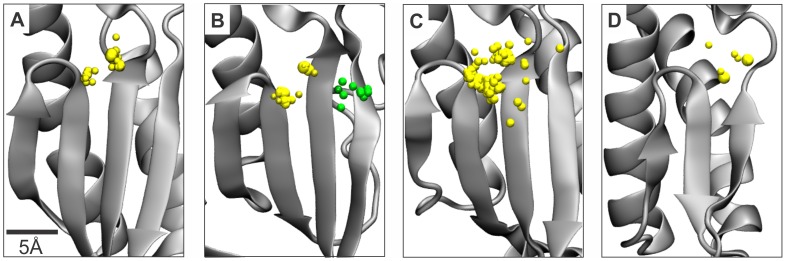
Positions of cations in the palm domain of hand-shaped DNA-dependent polymerases. The yellow spheres indicate catalytic ions, and the green spheres indicate cations that are positionally distinct from the catalytic cations. Structural alignments of (A) A family DdDps, (B) B family DdDps, (C) Y family DdDps, and (D) DdRps. The aligned cations are depicted with the polypeptide chain of the catalytic region from (A) *Bacillus stearothermophilus* DdDp (PDBid: 2BDP), (B) bacillus phage φ29 DdDp (PDBid: 2PYJ), (C) human DNA polymerase κ (PDBid: 2OH2), and (D) enterobacteria phage T7 DdRp (PDBid: 1S76).

In the PDB, 190 hand-shaped DdDp structures have been solved by X-ray crystallography and contain divalent cations close to the catalytic site. Alignment of these structures was carried out separately for members of DNA polymerase A (Fig. 4A; 51 structures), B (Fig. 4B; 25 structures), and Y families (Fig. 4C; 114 structures). Despite the large number of structures, metal ion ligands were not observed in positions corresponding to the non-catalytic ion-binding sites of vRdRps in any of the hand-shaped DdDps (Fig. 4). However, two DdDp B family members (escherichia phage RB69 gp43 and DdDp δ of *S. cerevisiae*) had 10 structures with ions at an approximate 6-Å distance from the catalytic site (Fig. 4B), positions clearly distinct from both non-catalytic ion-binding sites in vRdRps (Fig. 2A) and catalytic ions. As observed in vRdRps (Fig. 3), the third ion in three B family DdDp structures was coordinated by the conserved catalytic aspartate of motif A. Otherwise, the coordinating amino acids were dissimilar (see Table S3). Interestingly, a negatively charged amino acid located in the β-sheet adjacent to motif A and interacting with the third ion was highly conserved among members of the DdDp B family (corresponding E686 in the DdDp of phage RB69 and E802 in the DdDp δ of *S. cerevisiae*) [34].

The number of known hand-shaped DdRp structures is limited. In PDB, there are two enterobacteria phage T7 DdRp structures (PDBid: 1S76 and 1S77) and two enterobacteria phage N4 DdRp structures (PDBid: 3Q22 and 3Q23) that have ions near the catalytic site. The observed ions were catalytic ions in all four structures. Consequently, none of the currently available DdRp structures have metal ion ligands bound to locations resembling the positions of the non-catalytic ions in vRdRps (Fig. 4D).

### Structural Constraints for the Identification of Non-catalytic Ions in vRdRps

It was not possible to identify any sequence-based patterns that would allow the definition of the non-catalytic ion-binding sites in vRdRps (Fig. 3). Consequently, we decided to describe geometrical constraints that could be used for the identification of RdRp structures that contain non-catalytic ions.

Initially, we used two spheres, each with a definite radius and center (measuring point), and evaluated the presence of ions at the intersection of the spheres. As a starting point, the α-carbon of the first conserved aspartic acid of the GDD sequence in motif C (defined here as D1) was selected as measuring point one, and the α-carbon of the conserved aspartic acid in motif A (D2) was defined as measuring point two. These positions were selected as they can be unequivocally identified in all hand-shaped polymerases. Subsequently, the radii of the spheres and the positions of the measuring points were optimized independently for one-Ångström (range, 2 to 12 Å) and one-amino acid increments, respectively. This approach allowed the identification of constraints that enabled the recognition of all non-catalytic ion-containing RdRp structures (listed in Table 2). However, a few false-positive structures containing catalytic ions were also obtained. Consequently, a third measuring point was included near motif A, and all structures with ions within the third sphere were rejected. The position of measuring point three and the radii (range 0 to 12 Å) of the spheres was optimized as described above until all structures containing only catalytic ions were rejected without elimination of any of the structures containing non-catalytic ions. The constraints were as follows: measuring point one: D1+1 residue, radius 6 Å; measuring point two: D2-1 residue, radius 8 Å; measuring point three: D2+4 residues, radius 9 Å (see Fig. S1). These geometrical constraints correctly determined non-catalytic ions for RT structure 1N6Q and rejected all ions in the DdDp structures.

### Identity of the Non-catalytic Ions in vRdRps

The ions bound to the non-catalytic ion-binding sites in vRdRps were annotated as Mg^2+^ (15 structures) or Mn^2+^ (13 structures) (Table 2). A Ca^2+^ ion was described in three vRdRp structures, and a Lu^2+^ ion was identified in a single structure of rabbit hemorrhagic virus polymerase (PDBid: 1KHV). Depending on the structure, either Mg^2+^ or Ca^2+^ was observed at the position of the non-catalytic ion in the RdRps of West Nile virus and enterobacteria phage Qβ, and either Mg^2+^ or Mn^2+^ was observed in the RdRps of the Norwalk virus and pseudomonas phage φ6 (see Table S1). Based on these annotations, the identity of the bound ion did not appear to be dependent on either the viral family or species.

This finding raises a question regarding the type of bound ion *in vivo*. In φ6 RdRp, Mn^2+^ is considered the natural ion [16] that can be replaced by Mg^2+^ in the presence of 10 mM MgCl_2_ [17]. Consequently, the crystallization conditions for vRdRp structures with bound Mg^2+^, Mn^2+^, Ca^2+^, Lu^2+^, Sm^3+^, or no ion in the non-catalytic or catalytic ion-binding site were studied (see Table S1). Such analyses, carried out previously by Wright et al. [17] for a few viral RdRp structures, were extended here (see Table S1) to cover all currently available RdRp structures. In 56 vRdRp structures having either catalytic or non-catalytic ions, the identity of the bound ion(s) appeared to depend on the ionic conditions applied during the crystallization of the vRdRp. Nevertheless, the addition of ions did not always result in the detection of these ions in the final structure (37 vRdRp structures). Interestingly, in the mammalian reovirus RdRp structure (PDBid: 1MWH), a Mn^2+^ ion was bound to the non-catalytic ion-binding site, although no ion was added during the preparation of the crystals.

## Discussion

Polymerases are a large and diverse group of enzymes, but only a few of them have been structurally characterized [35]. In this study, we focused on a group of small, single subunit, hand-shaped polymerases [36]–[38], which included viral RdRps, RTs, DdRps, DdDps, and cellular DdDps. The analyses reported here propose that a common divalent cation-binding site, at an approximate 6-Å distance from the positions of the catalytic ions, exists in the polymerases of many dsRNA and positive-strand ssRNA viruses (Table 2 and Fig. 2B). Two DNA polymerase B family members also had ions at a 6-Å distance from the catalytic site, but the position differed from the non-catalytic ion-binding site in vRdRps. The non-catalytic ions, although proximal, could be readily distinguished from catalytic ions by a structural alignment of the palm region. To identify polymerases with bound non-catalytic ions, we describe simple distance-based constraints (see Fig. S1) that allow a more automated detection of such structures.

The presence of non-catalytic ions did not correlate with the initiation mechanism applied by vRdRps; bound ions were observed in vRdRps applying both *de novo* initiation (*Cystovirus*, *Orthoreovirus*, *Flavivirus*, and *Allolevivirus* genera; Table 2) and primer-dependent initiation mechanisms (*Lagovirus*, *Norovirus*, *Aphthovirus*, and *Enterovirus* genera; Table 2). In addition, there did not appear to be any specific stage of RNA polymerization during which the ion was present (see Table S1 and Fig. 2B).

Although a non-catalytic ion was not present in every available high-resolution vRdRp structure, it is still possible that binding sites exist in all or most vRdRps: i) There was no distinct family of viruses that did not have a bound ion (Table 2), ii) empty and ion-bound vRdRp structures were observed, even within a single viral species (see Table S1), and iii) the structural similarity of this site in all structurally characterized vRdRps was evident. In addition, the number vRdRp structures containing non-catalytic ions is comparable to the number of structures containing catalytic ions (32 versus 33 structures). Thus, the fact that non-catalytic ions have been detected in only a few RdRp structures most likely reflects technical issues during X-ray crystallography (see below). There is also biochemical evidence for the presence of a single divalent cation-binding site in hepatitis C virus RdRp, although numerous structural analyses of this vRdRp have not identified such a site [39]. Interestingly, an HIV RT structure also contained a bound Mg^2+^ ion at a similar structural position (Fig. 2C), suggesting that viral RTs may share this structural feature with vRdRps and that non-catalytic ion-binding sites may be common across all RNA-directed hand-shaped polymerases.

The common features of non-catalytic ion-binding sites of vRdRps and HIV RT found in this study are i) an approximate 4- to 6-Å distance from the position of catalytic ion A (Table 2), ii) the palm domain location between the β-strands of motifs A and C (Fig. 2A and 2C), and iii) the coordination of the bound ion by both an amino acid residue located within three residues upstream of the conserved aspartate of motif A (8 polymerases out of 10; Fig. 3) and an amino acid residue located at the position of the second aspartate of the GDD sequence (or its variant) within motif C (Fig. 3). Importantly, this second position of the GDD sequence is invariantly occupied by an aspartate in all RTs and RdRps of positive-strand ssRNA and dsRNA viruses, except for birnaviruses, which have an asparagine at an equivalent structural position (see below) [9], [10], [13]. In the DNA polymerase A and Y families, a glutamic acid occupies this position and is located next to the conserved aspartate residue to coordinatethe catalytic ions. Even though the physiochemical properties of aspartate are very similar to glutamate, divalent metal ion ligands bound to the position corresponding to the non-catalytic ion-binding site of vRdRps and HIV RT have not been observed in DNA polymerase A or Y families, despite the availability of numerous structures (see Fig. 4A and C, and Table S3). This finding suggests that the aspartate residue at this position is required for the binding of non-catalytic ions. Such an observation implies that the conservation of aspartate at this site in vRdRps and RTs may have arisen from the requirement to bind a third divalent cation proximal to the catalytic site and its fundamental role in nucleic acid polymerization catalyzed by this group of polymerases. Additionally, aspartate coordinates Mg^2+^, Ca^2+^, and Mn^2+^ more frequently than does glutamate [40].

Previous biochemical analyses of vRdRps and RTs support the importance of the second aspartate in the GDD sequence of motif C in the catalysis of viral RNA polymerization. Amino acid substitutions at this site significantly reduce the overall activity of vRdRps or entirely abolish RNA synthesis [41]–[45]. However, the effects of such a mutation in the poliovirus RdRp and HIV RT are suppressed in the presence of Mn^2+^ ions [44], [46]. This is in line with the observed effects of amino acid substitutions of distinct non-catalytic ion-coordinating amino acids (glutamic acid 491; see Table S1) in the pseudomonas phage φ6 RdRp [16]. Interestingly, the overall activity of the birnavirus RdRp is increased by mutating the ADN sequence of motif C to GDD [13]. Consequently, it has been proposed that birnaviruses may have maintained this ADN sequence to control their growth [13].

The present knowledge on the role of the third ion in B family DdDps is limited; thus, it is not possible to conclude whether this ion has a similar function as non-catalytic ions in vRdRps. However, mutations of the amino acid coordinating the third ion in gp43 of phage RB69 (residue E686A) result in similar phenotypes as mutations in the second aspartate of the GDD sequence in the poliovirus vRdRp and HIV RT [44], [46], [47]; the activity of these enzymes can be restored only in the presence of Mn^2+^ ions. In the DdDp δ of *S.cerevisiae*, mutations in amino acids coordinating the third ion reduce the efficiency of nucleotide incorporation [48]. Interestingly, the correct positioning of nucleotides at the catalytic site is also abolished in the RdRp of pseudomonas phage φ6 in the absence of non-catalytic Mn^2+^ ions [16].

The conserved aspartate of motif A interacts with non-catalytic ions in several vRdRps and in the HIV RT (Fig. 3). Therefore, it has been proposed that the side chain of this residue is able to adopt two different conformations, depending on the specific RNA polymerization step [49]. This would exclude the simultaneous presence of catalytic ions A and B and non-catalytic ions. However, there are three RdRp structures with three metal ions proximal to the catalytic site (Fig. 2B, panel II and VIII). Thus, it is possible that the binding patterns of the divalent metal ions in different vRdRps have slightly diverged or that the non-catalytic ions can be differentially coordinated at specific stages of the reaction.

Specific crystallization methods appear to affect the existence and type of bound ions at the non-catalytic ion-binding site. The presence of an inhibitory compound significantly reduces the probability of non-catalytic ion detection (Table 1), and the identity of the bound ion correlates with the ionic composition of the crystallization buffer (see Table S1). The identification of small ligands (e.g., ions) and the determination of their specific type from the electron density maps derived from X-ray diffraction data are technically demanding, especially if the resolution is moderate. Observable differences between different metal ions are small; consequently, there is the possibility of mistaken identities in published ion types. Such cases are likely to be biased towards annotations that reflect the ionic conditions used during crystallization experiments (see Table S1), overemphasizing Mg^2+^ over other ions.

There is only a single vRdRp structure in the PDB in which a bound non-catalytic ion is observed, although divalent cations were not included during the crystallization procedure (reovirus RdRp structure PDBid: 1MWH). Similar structures have also been described for pseudomonas phage φ6 [16]. In both cases, the bound ion was annotated as Mn^2+^. Moreover, the single divalent cation-binding site in the hepatitis C virus RdRp has a higher binding affinity for Mn^2+^ than Mg^2+^ [39]. Consequently, it is possible that Mn^2+^ is the natural ion at this site, which would explain the Mn^2+^ dependence of RNA polymerization described for many vRdRps [20]–[28], [50], [51].

Although divalent cations (e.g., Mg^2+^, Mn^2+^, Ca^2+^, and Lu^2+^) were present during crystallization, an ion was detected at the non-catalytic ion-binding site in only 32 out of 92 structures (Table 1 and Table S1). This observation likely reflects i) the difficulty in soaking ionin vRdRp crystals (i.e., catalytic ions were detected in only 33 structures, although the phenomenon is considered universal), ii) the predominant use of Mg^2+^ ions that may not optimally bind to the non-catalytic ion-binding site [17], iii) problems in the identification of small molecule ligands from the electron density maps, or iv) incomplete annotations (i.e., focus on the identification of a drug compound). However, it is possible that some vRdRps, in fact, do not have a functional non-catalytic ion-binding site, although viruses within the same family have such a feature.

It can be argued that the observed ions at the proposed non-catalytic ion-binding site reflect non-physiological conditions represented as crystallographic artifacts. Although possible in some cases, we consider that the presence of an ion at a similar position in 32 independent vRdRp structures produced using diverse crystallographic conditions makes such an assumption highly unlikely. Additionally, available biochemical and mutant data [41], [42], [44], [45] support the true existence of the ion-binding site and its biochemical significance.

The evident functional and structural similarities in the contemporary single subunit, hand-shaped polymerases suggest that these polymerases share a common origin. On the basis of the RNA world model, it has been hypothesized that RdRp is the most ancient form of nucleic acid polymerase and that ancestral RTs appeared as RdRp mutants with different substrate specificities [52]. DdRps likely evolved from RdRps that asymmetrically expressed positive-sense RNA from double-stranded nucleic acid molecules, like current vRdRps, whereas DdDps are possible descendants of ancestral RTs [52], [53]. The existence of a common non-catalytic ion-binding site in different viral polymerases (Table 2) implies that such a binding site has most likely been present in a primordial nucleic acid polymerase (an RdRp) from which contemporary RdRps and RTs originated; it is unlikely that such a property would have been separately acquired in each of the polymerases. Thus, it may be hypothesized that an RNA-dependent polymerase with a Mn^2+^-specific non-catalytic ion-binding site evolved in the ancient ocean in which the concentration of Mn^2+^ was substantially higher than it is today [54], [55] and that the specificity for this cation has been retained in at least some of the present RNA-dependent polymerases. DNA-dependent polymerases have probably lost the requirement to bind a third metal ion and have replaced the second aspartate of the GDD sequence with glutamate. Likewise, during evolution, some vRdRps may have lost their functional non-catalytic ion-binding site. An alternative, but unlikely, hypothesis for the origin of the non-catalytic ion-binding site is that the ability to bind a third divalent ion was acquired by an RNA-dependent polymerase at a later stage of evolution, after the differentiation of distinct polymerases, due to extensive horizontal exchange that allowed this property to spread among eukaryotic and prokaryotic RNA viruses.

Recent studies on the pseudomonas phage φ6 RdRp propose that the non-catalytic ion has an essential role in RNA polymerization both as an effector of the dynamic properties of the polymerase [17] and as a cofactor for the correct positioning of substrates for catalysis [16]. Additional biochemical and structural studies on different viral RdRps and RTs must be carried out to obtain further insights into the roles of non-catalytic ions in RNA-directed nucleic acid polymerization and to reveal possible functional conservation. The conventional two metal ion mechanism [12] for polymerases may need to be modified if the requirement for a third ion in catalysis can be established. Furthermore, high-resolution structural information of additional polymerases, especially RdRps of negative-strand ssRNA viruses, is required to present further conclusions on the conservation of the non-catalytic ion-binding site. As the non-catalytic ion-binding site appears to be a characteristic feature only of viral polymerases, it is a potential target for broad-spectrum antiviral drugs.

## Supporting Information

Figure S1 The geometrical constraints for the identification of hand-shaped polymerase structures with bound non-catalytic ions. A magnified view of the catalytic site from a hand-shaped polymerase (pseudomonas phage φ6; PDBid:1HI0) showing the conserved β-sheets in motifs A and C. The lined volume depicts the region in which the non-catalytic ion is located in all currently identified RdRp and RT structures. This volume can be defined using three spheres: I, II, and III (shown in blue, green, and mauve, respectively). The accepted non-catalytic ion-containing structures are ones in which the ions 1) are inside the intersection volume of spheres I and II, and 2) are not inside the volume of sphere III. The optimized centers of the spheres are defined by the location of the conserved aspartate residues in motifs C (D1) and A (D2), and the optimized radii are measured from an α-carbon of the indicated residues, which are shown as colored balls within the polypeptide chain of the polymerase.
(TIF)

Table S1 The RNA polymerase structures of the ssRNA viruses and dsRNA viruses. The PDBids of the analyzed RNA virus polymerase structures are given. In addition, resolution, ligands, crystallization conditions, presence of a template, a primer or mutations, observed ions close to the catalytic site and their coordinating amino acids are provided for each structure.
(PDF)

Table S2 The RdRp protein sequences used in the multiple alignment. The family name, species and the NCBI access number are given.
(PDF)

Table S3 The reverse transcriptase and DNA-dependent polymerase structures. The PDBids of the analyzed reverse transcriptses and DNA-dependent polymerases are given. In addition, species, resolution, ligands, mutations, observed ions close to the catalytic site and their coordinating amino acids are provided for each structure.
(PDF)
