# Supplementary figures and images for: Evidence for a Non-Catalytic Ion-Binding Site in Multiple RNA-Dependent RNA Polymerases
Source: PLoS One. 2012 Jul 11;7(7):e40581. doi: 10.1371/journal.pone.0040581 (PMC3394715; doi:10.1371/journal.pone.0040581)

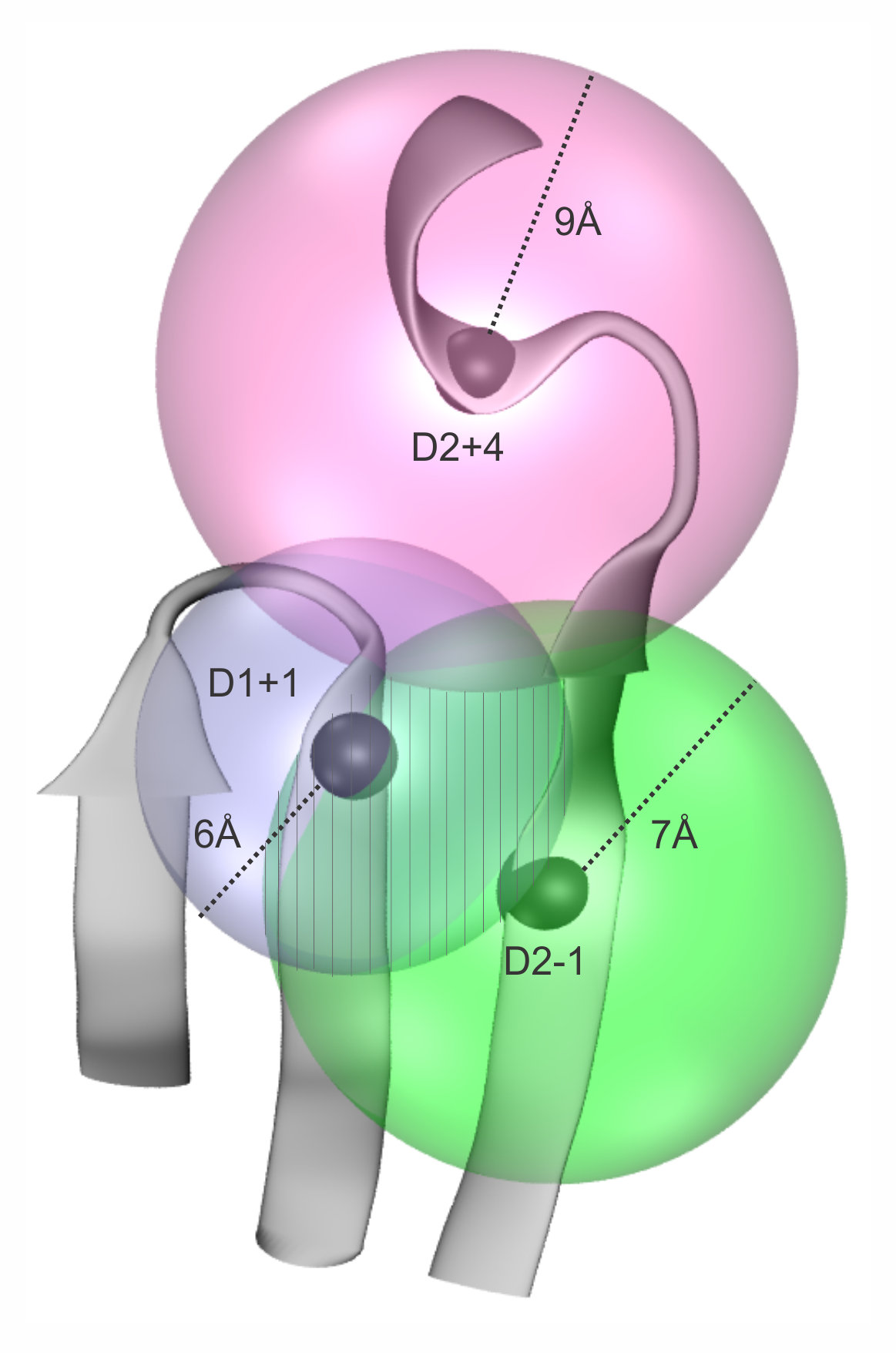

Supplement: Figure S1 — The geometrical constraints for the identification of hand-shaped polymerase structures with bound non-catalytic ions. A magnified view of the catalytic site from a hand-shaped polymerase (pseudomonas phage φ6; PDBid:1HI0) showing the conserved β-sheets in motifs A and C. The lined volume depicts the region in which the non-catalytic ion is located in all currently identified RdRp and RT structures. This volume can be defined using three spheres: I, II, and III (shown in blue, green, and mauve, respectively). The accepted non-catalytic ion-containing structures are ones in which the ions 1) are inside the intersection volume of spheres I and II, and 2) are not inside the volume of sphere III. The optimized centers of the spheres are defined by the location of the conserved aspartate residues in motifs C (D1) and A (D2), and the optimized radii are measured from an α-carbon of the indicated residues, which are shown as colored balls within the polypeptide chain of the polymerase. (TIF) [file pone.0040581.s001.tif]
