# Supplementary material for: Evidence for a Non-Catalytic Ion-Binding Site in Multiple RNA-Dependent RNA Polymerases
Source: PLoS One. 2012 Jul 11;7(7):e40581. doi: 10.1371/journal.pone.0040581 (PMC3394715; doi:10.1371/journal.pone.0040581)
Supplement: Table S1 — The RNA polymerase structures of the ssRNA viruses and dsRNA viruses. The PDBids of the analyzed RNA virus polymerase structures are given. In addition, resolution, ligands, crystallization conditions, presence of a template, a primer or mutations, observed ions close to the catalytic site and their coordinating amino acids are provided for each structure. (PDF) [file pone.0040581.s002.pdf]

Supporting information Table S1. The RNA polymerase structures of the ssRNA viruses and dsRNA viruses

Positive-sense ssRNA viruses

Family	Genus	Species	PDB ID	Resolution (Å)	Catalytic ion A[1]	Catalytic ion B	Non-catalytic ion, C	Ligands[2]	Stage of replication	Metal interactions	Presence of template and primer, mutations	Purification and storage	Crystallization
Caliciviridae	*Lagovirus*	rabbit haemorrhagic virus	1KHV	2.50	----	----	Lu^{2+} (B chain)	----	----	C: D355, G356, L249	----	column wash: 0.25 mM EDTA storage: 10 mM $MgCl_2$	crystallization: 5 mM $LuCl_2$
		rabbit haemorrhagic virus	1KHW	2.70	Mn^{2+}	Mn^{2+}	----	----	----	A: D354, D355, D250<br>B: D354, T251, D250	----	column wash: 0.25 mM EDTA storage: 10 mM $MgCl_2$	crystallization: 50 mM $Mg(NO_3)_2$, Soaking: 18 mM $MnCl_{2,}$ 10 mm 3′-deoxy-ATP
	Norovirus	Norwalk virus	1SH0	2.17	----	----	----	----	----	----	-----	column equilibration and elution: 0.25 mM EDTA	----
		Norwalk virus	1SH2	2.30	----	----	----	----	----	----	----	column equilibration and elution: 0.25 mM EDTA	----
		Norwalk virus	1SH3	2.95	----	----	Mg^{2+}	----	----	A: D344, D240	----	column equilibration and elution: 0.25 mM EDTA	crystallization: 20 mM $MgSO_4$
		Norwalk virus	2B43	2.30	----	----	----	----	----	----	----	Not information	Not information
		Norwalk virus	3BSN	1.80	Mn^{2+}	Mn^{2+}	----	GOL, N5C	----	A: D343, D344, D242, N5C<br>B: D343, Y243, D242, N5C	primer, template	column equilibration: 1 mM EDTA column elutation and dialyzation: 0.5 mM EDTA	crystallization: 0.97 mM NCT, 0.92 mM $MgCl_2$, 2.31 mM $MnCl_2$
		Norwalk virus	3BSO	1.74	Mn^{2+}	Mn^{2+}	----	GOL, CTP	----	A: D343, D344, D242, CTP<br>B: D343, Y243, D242, CTP	primer, template	column equilibration: 1 mM EDTA column elutation and dialyzation: 0.5 mM EDTA	crystallization: 0,97 mM CTP; 0,92 mM $MgCl_2$; 2,31 mM $MnCl_3$
		Norwalk virus	3H5Y	1.77	Mn^{2+}	Mn^{2+}	Mn^{2+}	GOL, CTP	----	A: D343, D344, D242, CTP<br>B: D343, Y243, D242, CTP<br>C: D344, A241	primer, template	column equilibration: 1 mM EDTA column elutation and dialyzation: 0.5 mM EDTA	soaking: 1.7 mM $MgCl_{2,}$ 4.3, 6.0 or 13 mM $MnCl_{2,}$ 7 mM CTP, 7mM $MnCl_2$.
		Norwalk virus	3H5X	1.77	Mn^{2+}	Mn^{2+}	Mn^{2+}	GOL, CSG	----	A: D343, CSG<br>B: D343, Y243, CSG<br>C: D344, A241	primer, template	column equilibration: 1 mM EDTA column elutation and dialyzation: 0.5 mM EDTA	soaking: 1.7 mM $MgCl_2$ and either 4.3, 6.0 or 13 mM $MnCl_{2,}$ 7 mM CTP and 7mM $MnCl_2$.
	Sapovirus	Sapporo virus	2CKW	2.32	----	----	----	----	----	----	----	dialyzation: 5 mM $MgCl_2$	----
		Sapporo virus	2UUW	2.76	----	----	----	----	----	----	D347G, D348G	----	No information
		Sapporo virus	2UUT	2.40	----	----	----	----	----	----	D346G	----	No information
		Sapporo virus	2WK4	2.98	----	----	----	GOL	----	----	D347G, D348G	----	No information
Flaviviridae	*Flavivirus*	Dengue virus	2J7U	1.85	----	----	Mg^{2+}	Cl, PGE, Zn^{2+}	----	C: D664	----	dialyzation, eluation, gel filtration: 1 mM EDTA	crystallization: 1 mM EDTA, qualification: $MgSO_4$ or $Mg(CH_3COO)_2$ at concentrations of 0.005 to 0.2 M, soaking: 0.2 M $MgCl_2$
		Dengue virus	2J7W	2.60	----	----	----	GTP, PEG, Zn^{2+}	----	----	----	dialyzation, eluation, gel filtration:1 mM EDTA	crystallization: 1 mM EDTA, qualification: $MgSO_4$ or magnesium acetate at concentrations of 0.005 to 0.2 M, soaking: 40 mM $MgSO_4$, 10 mM 3′dGTP
		West Nile virus	2HCN	2.35	----	----	Ca^{2+}	Zn^{2+}	----	C: D536, D669	----	----	crystallization: 0.2 M calcium acetate
		West Nile virus	2HCS	2.50	----	----	----	Zn^{2+}	----	----	----	----	No information
		West Nile virus	2HFZ	2.50	----	----	Mg^{2+}	Zn^{2+}	----	C: D536, D669, A534	----	----	crystallization: 0.3 M $MgCl_2$
	Hepacivirus	hepatitis C virus	2XWH	1.80	----	----	----	15P, PEG	----	----	----	dialyzation: 1 mM EDTA	----
		hepatitis C virus	1C2P	1.90	----	----	----	MSE	----	----	----	----	----
		hepatitis C virus	1CSJ	2.80	----	----	----	MSE	----	----	----	the sample was concentrated to 1 mM EDTA	----
		hepatitis C virus	1GX5	1.70	Mn^{2+}	Mn^{2+}	----	GTP, Mn^{2+}	----	A: D318, D319, GTP<br>B: D318, T221, D220, GTP	----	----	soaking: 10mM rGTP and 11 mM $MnCl_2$

		hepatitis C virus	1GX6	1.85	Mn^{2+}	Mn^{2+}	----	UTP, Mn^{2+}	----	A:D318, D319, D220, UTP<br>B:D318, T221, D220, UTP	----	----	soaking: 10 mM rUTP and 11 mM $MnCl_2$
		hepatitis C virus	1NB4	2.00	----	----	----	---	----	----	----	bacteria pellet resuspension: 10 mM $MgCl_2$	cryoprotectant solution: 15 mM $MnCl_2$, 12.5 mM rNTP
		hepatitis C virus	1NB6	2.60	Mn^{2+}	Mn^{2+}	----	UTP	----	A: D318, D319, D220, UTP<br>B: D318, T221, D220, UTP	----	bacteria pellet resuspension: 10 mM $MgCl_2$	cryoprotectant: 15 mM $MnCl_2$, 12.5 mM rNTP
		hepatitis C virus	1NB7	2.90	Mn^{2+}	Mn^{2+}	----	----	----	A: -<br>B: F224,C223, T221	short template	bacteria pellet resuspension: 10 mM $MgCl_2$	cryoprotectant: 15 mM $MnCl_2$, 12.5 mM rNTP
		hepatitis C virus	1NHU	2.00	----	----	----	153	----	----	----	----	soaking: 10 mM $MgCl_2$
		hepatitis C virus	1NHV	2.90	----	----	----	154	----	----	----	----	soaking: 10 mM $MgCl_2$
		hepatitis C virus	1OS5	2.20	----	----	----	NH1	----	----	L47Q, F101Y, K114R	equilibration, storage: 5 mM $MgCl_2$	----
		hepatitis C virus	1QUV	2.50	----	----	----	----	----	----	R2963Q	----	No information
		hepatitis C virus	1YUY	1.90	----	----	----	SO_4	----	----	----	----	----
		hepatitis C virus	1YV2	2.50	----	----	----	GOL, SO_4	----	----	----	----	----
		hepatitis C virus	1YVF	2.50	----	----	----	Cl^-, GOL, PH7, PO_4	----	----	----	----	No information
		hepatitis C virus	1YVX	2.00	----	----	----	IPC, SO_4	----	----	----	----	----
		hepatitis C virus	1YVZ	2.20	----	----	----	JPC, SO_4	----	----	----	----	----
		hepatitis C virus	1Z4U	2.80	----	----	----	Cl^-, GOL, PH9, PO_4	----	----	----	----	No information
		hepatitis C virus	2AWZ	2.15	----	----	----	5H, SO_4	----	----	V499A, S506N, Q514R, T520I, P540A, S543G, V552T, S563G, L564V, R566H	column equilibration: 10 mM $MgCl_2$	----
		hepatitis C virus	2AX0	2.00	----	----	----	5X, SO_4	----	----	V499A, S506N, Q514R, T520I, P540A, S543G, V552T, S563G, L564V, R566H	column equilibration: 10 mM $MgCl_2$	----
		hepatitis C virus	2AX1	2.10	----	----	----	5EE, SO_4	----	----	499A, S506N, Q514R, T520I, P540A, S543G, V552T, S563G, L564V, R566H	column equilibration: 10 mM $MgCl_2$	----
		hepatitis C virus	2BRK	2.30	Mn^{2+}	Mn^{2+}	----	CMF	----	A: D318, D319, D220<br>B: D318, T221, D220	----	equilibration: 1 mM EDTA	soaking: 10 mM $MnCl_2$, cryprotectant: 5 mM $MnCl_2$
		hepatitis C virus	2BRL	2.40	Mn^{2+}	Mn^{2+}	----	POO	----	A: D318, D319, D220<br>B: D318, T221, D220	----	equilibration: 1 mM EDTA	soaking: 10 mM $MnCl_2$, cryoprotectant: 5 mM $MnCl_2$
		hepatitis C virus	2D3U	2.00	----	----	----	CCT	----	----	----	----	soaking: 10 mM $MgCl_2$
		hepatitis C virus	2D3Z	1.80	----	----	----	FIH	----	----	----	----	soaking: 10 mM $MgCl_2$
		hepatitis C virus	2D41	2.10	----	----	----	SNH	----	----	----	----	soaking: 10 mM $MgCl_2$
		hepatitis C virus	2DXS	2.20	----	----	----	JTP	----	----	----	----	----
		hepatitis C virus	2FVC	2.00	----	----	----	888	----	----	----	----	No information
		hepatitis C virus	2GC8	2.20	----	----	----	885	----	----	----	----	No information
		hepatitis C virus	2GIQ	1.65	----	----	----	NN2	----	----	----	----	----
		hepatitis C virus	2GIR	1.90	----	----	----	NN3	----	----	----	----	----
		hepatitis C virus	2HAI	1.58	----	----	----	PFI	----	----	K114R, L47Q, F101Y	----	No information
		hepatitis C virus	2HWH	2.30	----	----	----	RNA(inhibitor)	----	----	----	----	----
		hepatitis C virus	2HWI	2.00	----	----	----	VRX	----	----	----	----	----
		hepatitis C virus	2I1R	2.20	----	----	----	VXR	----	----	----	----	No information
		hepatitis C virus	2IJN	2.20	----	----	----	221	----	----	----	----	----
		hepatitis C virus	2JC0	2.20	----	----	----	699	----	----	----	Column equilbration and wash, and storage: 0.1 mM EDTA	No information
		hepatitis C virus	2JC1	2.00	----	----	----	698	----	----	----	----	No information
		hepatitis C virus	2O5D	2.20	----	----	----	VR1	----	----	----	----	----
		hepatitis C virus	2QE2	2.90	----	----	----	452	----	----	----	----	----
		hepatitis C virus	2QE5	2.60	----	----	----	617	----	----	----	----	----
		hepatitis C virus	2WCX	2.00	Mn^{2+}	Mn^{2+}	----	VGC	----	A: D318, D319, D220<br>B: D318, T221, D220	----	----	soaking: 10 mM $MnCl_2$
		hepatitis C virus	2WHO	2.00	Mn^{2+}	Mn^{2+}	----	VGI	----	A: D318, D319, D220<br>B: D318, T221, D220	----	----	soaking: 10 mM $MnCl_2$
		hepatitis C virus	2WRM	1.95	----	----	---	QQ3	----	----	----	----	No information

		hepatitis C virus	2XHU	2.29	----	----	----	SO_4	----	----	----	dialyzation: 1 mM EDTA	crystallization: 0.2 M $MgSO_4$ soaking:10 mm $MgCl_2$, 10 mm GTP
		hepatitis C virus	2XHV	1.90	----	Mg^{2+}	----	SO_4	----	B: D318, T221, D220	S556K	dialyzation: 1 mM EDTA	crystallization: 0.2 M $MgSO_4$ soaking:10 mM $MgCl_2$, 10 mM GTP
		hepatitis C virus	2XHW	2.66	----	----	----	----	----	----	----	dialyzation: 1 mM EDTA	----
		hepatitis C virus	2XI2	1.80	----	----	----	SO_4	----	----	S556K	----	crystallization: 2.5 mM $MgCl_2$, 1 mM EDTA, 0.5 mM GTP
		hepatitis C virus	2XI3	1.70	Mg^{2+} (?)	Mg^{2+} (?)	----	GTP	----	A: GTP<br>B:T221, D220, GTP	S556K	----	crystallization: 2.5 mm $MgCl_2$, 1 mm EDTA, soaking : 10 mm $MgCl_2$
		hepatitis C virus	2XWY	2.53	Mn^{2+}	Mn^{2+}	----	1B8	----	A: D318, D319, D220<br>B: D318, T221, D220	----	----	soaking: 10 mM $MnCl_2$, 5 mM $MnCl_2$
		hepatitis C virus	2XXD	1.88	----	----	----	PO_4	----	----	----	dialyzation: 1 mM EDTA	----
		hepatitis C virus	2XYM	1.77	----	----	----	PO_4	----	----	I405	dialyzation: 1 mM EDTA	----
		hepatitis C virus	2ZKU	1.95	----	----	----	ACY, GOL	----	----	----	----	No information
		hepatitis C virus	3BR9	2.30	----	----	----	DEY	----	----	Q544R	----	soaking: 10 mM $MgCl_2$
		hepatitis C virus	3BSA	2.30	----	----	----	1PD	----	----	Q544R	----	soaking: 10 mM $MgCl_2$
		hepatitis C virus	3BSC	2.65	----	----	----	2PD	----	----	Q544R	----	soaking: 10 mM $MgCl_2$
		hepatitis C virus	3CDE	2.10	----	----	----	N3H	----	----	R544Q	----	soaking: 10 mM $MgCl_2$
		hepatitis C virus	3CIZ	1.87	----	----	----	SX1, Zn^{2+}	----	----	----	----	No information
		hepatitis C virus	3CJ0	1.90	----	----	----	SX2	----	----	----	----	No information
		hepatitis C virus	3CJ2	1.75	----	----	----	Ni^{2+}, SX3	----	----	----	----	No information
		hepatitis C virus	3CJ3	1.87	----	----	----	Ni^{2+}, SX4	----	----	----	----	No information
		hepatitis C virus	3CJ4	2.07	----	----	----	Ni^{2+}, SX5	----	----	----	----	No information
		hepatitis C virus	3CJ5	1.92	----	----	----	SX6	----	----	----	----	No information
		hepatitis C virus	3CO9	2.10	----	----	----	3MS	----	----	Q544R	----	soaking: 10 mM $MgCl_2$
		hepatitis C virus	3CSO	2.71	----	----	----	XNI	----	----	----	----	crystallization: 100 mM Mg salts
		hepatitis C virus	3CWJ	2.40	----	----	----	321	----	----	R544Q	----	soaking: 10 mM $MgCl_2$
		hepatitis C virus	3CVK	2.31	----	----		N34	----	----	Q544R	----	soaking: 10 mM $MgCl_2$
		hepatitis C virus	3D28	2.30	----	----	----	B34	----	----	R2963Q	----	soaking: 10 mM $MgCl_2$
		hepatitis C virus	3D5M	2.20	----	----	----	4MS	----	----	R544Q	----	soaking: 10 mM $MgCl_2$
		hepatitis C virus	3E51	1.90	----	----	----	N35	----	----	R544Q	----	soaking: 10 mM $MgCl_2$
		hepatitis C virus	3FQK	2.20	----	----	----	79Z	----	----	---	----	----
		hepatitis C virus	3FQL		----	----	----	79Z	----	----	----	----	----
		hepatitis C virus	3FRZ	1.86	----	----	----	AG0, AG6, BME	----	----	K114R, L47Q, F101Y, V59D	----	No information
		hepatitis C virus	3G86	2.20	----	----	----	Ni^{2+}, T18	----	----	----	----	No information
		hepatitis C virus	3GNV	2.75	----	----	----	GOL, XNZ	----	----	----	----	----
		hepatitis C virus	3GNW	2.39	----	----	----	Cl^-, XNC	----	----	----	----	----
		hepatitis C virus	3GOL	2.85	Mg^{2+} (?)	----	----	XND	----	A: T221,D220	----	----	Not information
		hepatitis C virus	3GSZ	1.90	----	----	----	----	----	----	----	DNAase tratment: $MgCl_2$ (10 mM)	----
		hepatitis C virus	3GYN	2.15	----	----	----	B42	----	----	----	No information	soaking: 10 mM $MgCl_2$
		hepatitis C virus	3H2L	1.90	----	----	----	YAK	----	----	R544Q	No information	soaking: 10 mM $MgCl_2$
		hepatitis C virus	3H59	2.10	----	----	----	H59	----	----	----	----	----
		hepatitis C virus	3H5S	2.00	----	----	----	H5S	----	----	----	----	----
		hepatitis C virus	3H5U	1.95	----	----	----	H5U	----	----	----	----	----
		hepatitis C virus	3H98	1.90	----	----	----	B5P, GOL	----	----	----	----	No information
		hepatitis C virus	3HHK	1.70	----	----	----	77Z	----	----	----	----	----
		hepatitis C virus	3HKW	1.55	----	----	----	GOL, IX6, SO_4	----	----	----	----	----
		hepatitis C virus	3HKY	1.90	----	----	----	Cl^-,IX6, SO_4	----	----	----	----	----
		hepatitis C virus	3HVO	2.00	----	----	----	VG1	----	----	S5T	----	----
		hepatitis C virus	3I5K	2.20	----	----	----	PO_4	----	----	----	dialyzation: 1 mM EDTA	----
		hepatitis C virus	3IGV	2.60	----	----	----	B80	----	----	R544Q	No information	soaking: 10 mM $MgCl_2$
		hepatitis C virus	3LKH	2.05	----	----	----	LT6	----	----	----	----	No information
		hepatitis C virus	3MF5	2.00	----	----	----	GOL, HJZ	----	----	----	----	No information
		hepatitis C virus	3MWV	2.20	----	----	----	----	----	----	----	----	----
		hepatitis C virus	3MWW	2.80	----	----	----	BIW, SO_4	----	----	----	----	----
	Pestivirus	bovine viral diarrhea virus	1S48	3.00	----	----	----	----	----	----	----	----	----
		bovine viral diarrhea virus	1S49	3.00	----	----	----	----	----	----	----	----	----

		bovine viral diarrhea virus	1S4F	3.00	----	----	----	----	----	----	----	----	----
		bovine viral diarrhea virus	2CJQ	2.60	----	----	----	----	----	----	----	----	----
Leviviridae	*Allolevivirus*	enterobacteria virus Qβ	3AGP	2.80	----	----	Ca^{2+}	----	----	C: D968, D1054, E1089	----	----	crystallization: $C_4H_6CaO_4$
		enterobacteria virus Qβ	3AGQ	3.22	----	----	Mg^{2+}	----	----	C: D968, D1054	----	----	crystallization: $Mg(CH_3COO)_2$
		enterobacteria virus Qβ	3MMP	2.50	----	----	----	PXN	----	----	elongation factor Tu2 and Ts	----	----
Picornaviridae	*Aphthovirus*	foot and mouth disease virus	1U09	1.91	----	----	----	----	----	----	----	dialyzation: 1 mM EDTA	----
	,	foot and mouth disease virus	1WNE	3.00	----	----	Mg^{2+}	----	----	C: D238, V239, D240, D339	template, primer	dialyzation: 1 mM EDTA	crustallization: 2 mM $MgCl_2$
		foot and mouth disease virus	2F8E	2.90	----	----	----	U5P, Mg^{2+}, Mn^{2+}	----	----	----	dialyzation: 1 mM EDTA	crystallization: 2 mM UTP, 2 mM $MnCl_2$ and 2 mM $MgCl_2$
		foot and mouth disease virus	2D7S	3.00	----	----	----	----	----	----	VPg	dialyzation: 1 mM EDTA	crystallization: 2 mM $MnCl_2$
		foot and mouth disease virus	2EC0	2.75	----	----	Mg^{2+}	PPV	----	C: D238, T384, D339	template, primer	dialyzation: 1 mM EDTA	crystallization: 2 mM $MgCl_2$, soaking: 2 mM ATP, 2 mM $MnCl_2$
		foot and mouth disease virus	2E9Z	3.00	----	Mg^{2+} (?)	Mg^{2+}	PPV, UTP	----	B: UTP, Y241<br>C: D238, T384, D339	template, primer	dialyzation: 1 mM EDTA	crystallization: 2 mM $MgCl_2$; soaking: 2 mM $MnCl_2$; 2 mM UTP
		foot and mouth disease virus	2E9T	2.60	----	Mg^{2+} (?)	Mg^{2+}	PPV	----	C: T384, D339, D238	template, primer (modified 5FU)	dialyzation: 1 mM EDTA	crystallization 2 mM $MgCl_2$; soaking 2 mM $MnCl_2$ and 2 mM FUTP
		foot and mouth disease virus	2E9R	2.81	----	----	Mg^{2+}	RTP	----	C: D238, D339	template, primer	dialyzation: 1 mM EDTA	crystallization: 2 mM $MgCl_2$; soaking: 2 mM $MnCl_2$ and 2 mM RTP
		foot and mouth disease virus	3KOA	2.40	----	Mg^{2+} (?)	Mg^{2+}	DPO	----	B: Y241<br>C: D339, D240, D238, T384	two polyribonucleotides; M296I	dialyzation: 1 mM EDTA	crystallization: 2 mM $MgCl_2$; soaking: 2 mM $MnCl_2$, and 2 mM GTP
		foot and mouth disease virus	3KNA	2.80	----	----	Mg^{2+}	----	----	C: D238, D240, D339, T384	two polyribonucleotides; M296I	dialyzation: 1 mM EDTA	crystallization: 2 mM $MgCl_2$; soaking: 2 mM $MnCl_2$, and 2 mM GTP
		foot and mouth disease virus	3KMS	2.20	----	----	Mg^{2+}	----	----	C: D238, D240	two polyribonucleotides; G62S	dialyzation: 1 mM EDTA	crystallization: 2 mM $MgCl_2$; soaking: 2 mM $MnCl_2$, and 2 mM GTP
		foot and mouth disease virus	3KMQ	2.11	----	----	----	----	----	----	two polyribonucleotides; G62S	dialyzation: 1 mM EDTA	crystallization: 2 mM $MgCl_2$; soaking: 2 mM $MnCl_2$, and 2 mM GTP
		foot and mouth disease virus	3KLV	2.60	----	----	Mg^{2+}	----	----	C: D238, D339, T384	two polyribonucleotides; G62S M296I	dialyzation: 1 mM EDTA	crystallization: 2 mM $MgCl_2$; soaking: 2 mM $MnCl_2$, and 2 mM GTP
	Enterovirus	human enterovirus B (coxsackie virus)	3CDW	2.50	----	----	----	ACT, Cl^-, GOL, POP	----	----	VPg	----	incubation before crystallization: 2.5 mM 3′dUTP and 10 mM $MgCl_2$
		human enterovirus B (coxsackie virus)	3CDU	2.10	----	----	----	ACT, Cl^-, GOL, POP, SO_4	----	----	----	----	incubation before crystallization: 10 mM NTP and 10 mM MgCl2 or $MnCl_2$
		human enterovirus B (coxsackie virus)	3DDK	2.25	----	----	----	Na^+, SO_4	----	----	----	----	----
		human enterovirus C (poliovirus)	1RDR	2.40	----	----	Ca^{2+}	----	----	C: D233, D329	----	protein loading onto column, washing and elution: 0.1 mM EDTA	crystallization: 0.6 M $CaCl_2$; 0.2 mM EDTA harvested into and soaking: 1.0–1.2 M $CaCl_2$ and 0.2 mM EDTA
		human enterovirus C (poliovirus)	1TQL	2.30	----	----	----	ACY	----	----	G1A, L446D, R455D	protein loading onto column, washing and elution: 0.1 mM EDTA	----
		human enterovirus C (poliovirus)	1RAJ	2.35	----	----	----	----	----	----	L446A, R455D	protein loading onto column, washing and elution: 0.1 mM EDTA	----

		human enterovirus C (poliovirus)	1RA7	2.50	----	----	----	ACY, GTP	----	----	L446D, R455D	protein loading onto column, washing and elution: 0.1 mM EDTA	----
		human enterovirus C (poliovirus)	1RA6	2.00	----	----	----	ACY	----	----	L446D, R455D	protein loading onto column, washing and elution: 0.1 mM EDTA	----
		human enterovirus C (poliovirus)	2ILZ	2.50	Mn^{2+}	Mn^{2+}	----	ACY, GTP, Mn^{2+}, Na^{+}	----	A: D328<br>B: Y234, GTP	L446D, R455D	----	soaking: 10 mM GTP and 10 mM $MnCl_2$
		human enterovirus C (poliovirus)	2ILY	2.60	----	----	----	ACY, ATP, Na^{+}	----	----	L446D, R455D	----	soaking: 10 mM ATP and 10 mM $MgCl_2$
		human enterovirus C (poliovirus)	2IM3	2.60	Mn^{2+} (?)	Mn^{2+} (?)	----	ACY, Na^{+}, UTP	----	A: D328<br>B: D328, Y234, UTP	L446D, R455D	----	soaking: 10 mM UTP and 10 mM $MnCl_2$
		human enterovirus C (poliovirus)	2IM2	2.35	----	----	----	ACY, Na^{+}, UTP	----	----	L446D, R455D	----	soaking: 10 mM UTP and 10 mM $MgCl_2$
		human enterovirus C (poliovirus)	2IM1	2.50	Mn^{2+} (?)	Mn^{2+} (?)	----	ACY, CTP, Na^{+}, Mn^{2+}	----	A: D328<br>B:Y234, CTP	L446D, R455D	----	soaking: 10 mM CTP and 10 mM $MnCl_2$
		human enterovirus C (poliovirus)	2IM0	2.25	----	----	----	ACY, CTP, Na^{+}	----	----	L446D, R455D	----	soaking: 10 mM CTP and 10 mM $MgCl_2$
		human enterovirus C (poliovirus)	2IJF	3.00	----	----	----	----	----	----	G64S, L446D, R455D	0.5 mM EDTA	----
		human enterovirus C (poliovirus)	2IJD	3.40	----	----	----	SO4, Zn^{2+}	----	----	E55A, D58A, E63A, C147A, L629D, R638D	0.5 mM EDTA	----
		human enterovirus C (poliovirus)	3OLA	2.55	----	----	----	DCP, GOL, IPA, PEG, Zn^{2+}	elongation	----	L446D, template, product, nontemplate	column equilibration: 5 mM $MgCl_2$	soaking: 10 mM $MgCl_2$ and 5 mM CTP, 2′-dCTP, 3′-dCTP, or 2′,3′-ddCTP
		human enterovirus C (poliovirus)	3OLB	2.41	----	----	----	DCT, IPA, Zn^{2+}	elongation	----	L446D, template, product, nontemplate	column equilibration: 5 mM $MgCl_2$	soaking: 10 mM $MgCl_2$ and 5 mM CTP, 2′-dCTP, 3′-dCTP, or 2′,3′-ddCTP
		human enterovirus C (poliovirus)	3OL6	2.50	----	----	----	IPA, Zn^{2+}	elongation	----	L446D, template, product, nontemplate	column equilibration: 5 mM $MgCl_2$	soaking: 10 mM $MgCl_2$ and 5 mM CTP, 2′-dCTP, 3′-dCTP, or 2′,3′-ddCTP
		human enterovirus C (poliovirus)	3OL7	2.70	Mg^{2+}	Mg^{2+}	----	GOL, IPA, PEG, POP, Zn^{2+}	elongation	A: D328, D329, D233<br>B: D238, Y234, D233	L446D, template, product, nontemplate	column equilibration: 5 mM $MgCl_2$	soaking: 10 mM $MgCl_2$ and 5 mM CTP, 2′-dCTP, 3′-dCTP, or 2′,3′-ddCTP
		human enterovirus C (poliovirus)	3OL8	2.75	Mn^{2+}	Mn^{2+}	----	GOL, IPA, POP, Zn^{2+}	elongation	A: D328, D329, D233<br>B: D328, Y234, D233, POP	L446D, template, product, nontemplate	column equilibration: 5 mM $MgCl_2$	soaking: 10 mM $MnCl_2$ and 5 mM CTP, 2′-dCTP, 3′-dCTP, or 2′,3′-ddCTP
		human enterovirus C (poliovirus)	3OL9	2.25	----	----	----	IPA, POP, Zn^{2+}	elongation	----	L446D, template, product, nontemplate	column equilibration: 5 mM $MgCl_2$	soaking: 10 mM $MgCl_2$ and 5 mM CTP, 2′-dCTP, 3′-dCTP, or 2′,3′-ddCTP
		human rhinovirus A	1TP7	2.40	----	----	----	DMX, SO_4	----	----	----	----	----
		human rhinovirus A	1XR5	2.80	Sm^{3+} (?)	----	----	----	----	----	----	----	soaking: 0.25 mM $SmCl_3$
		human rhinovirus A	1XR6	2.50	----	----	----	K^{+}	----	----	----	----	crystallization: ATP analog AMPPNP: 2 mM $MnCl_2$ or $MgCl_2$
		human rhinovirus A	1XR7	2.30	----	----	----	----	----	----	----	----	crystallization: ATP analog AMPPNP: 2 mM $MnCl_2$ or $MgCl_2$

Double-stranded RNA viruses

Family	Genus	Species	PDB ID	Resolution (Å)	Catalytic ion A	Catalytic ion B	Non-catalytic ion, C	Ligands	Stage of replication	Metal interactions	Presence of template and primer, mutations	Purification and storage	Crystallization

Birnaviridae	*Avibirnavirus*	infectious bursal disease virus	2PGG	2.50	----	----	----	----	----	----	----	----	----
		infectious bursal disease virus	2QJ1	3.48	----	----	----	----	----	----	----	----	No information
		infectious bursal disease virus	2PUS	2.40	----	----	----	----	----	----	----	----	----
		infectious bursal disease virus	2R70	2.70	----	----	----	----	----	----	----	----	----
		infectious bursal disease virus	2R72	3.15	Mg^{2+} (?)	Mg^{2+} (?)	Mg^{2+}	----	----	A: N493, D402<br>B: D402, G420, L417<br>C: N403, D416, I415, S414	----	----	soaking: 2 mM $MgCl_2$ and 2 mM GTP
Cystoviridae	*Cystovirus*	pseudomonas phage φ6	1HI0	3.00	Mg^{2+} (?)	Mg^{2+} (?)	Mn^{2+}	GTP	initiation	A: GTP<br>B:D453, V325, GTP<br>C: D454, E491, A495	short template	elution from the column: 1 mM EDTA	crystallization: 8 mM $MgCl_2$, Soaking: 10 mM AMPPNP, AMPCPP or CTP (all in the presence of 2 mM $MnCl_2$)
		pseudomonas phage φ6	1HI8	2.50	----	----	Mg^{2+}	----	----	C: D454, E491, A495	----	elution from the column: 1 mM EDTA	crystallization: 8 mM $MgCl_2$
		pseudomonas phage φ6	1HI1	3.00	----	----	----	ATP	----	----	----	elution from the column: 1 mM EDTA	crystallization: 8 mM $MgCl_2$, Soaking: 10 mM AMPPNP, AMPCPP or CTP (all in the presence of 2 mM $MnCl_2$)
		pseudomonas phage φ6	1HHS	2.00	----	----	Mn^{2+}	----	----	C: D454, E491, A495	----	elution from the column: 1 mM EDTA	crystallization: 8 mM $MgCl_2$
		pseudomonas phage φ6	1HHT	2.90	----	----	Mn^{2+}	----	----	C: D454, E491, A495	template	elution from the column: 1 mM EDTA	crystallization: 8 mM $MgCl_2$; soaking: 2 mM $MnCl_2$
		pseudomonas phage φ6	1UVI	2.15	----	----	Mn^{2+}	----	----	C: D454, E491, A495	template	elution from the column: 1 mM EDTA	soaking: 40–60 mM GTP and 25 mM Mg^{2+} and Ca^{2+}; In the presence of 2 mM MnCl2
		pseudomonas phage φ6	1UVJ	1.90	----	----	Mn^{2+}	----	----	C: D454, E491, A495	ribonucleotide	elution from the column: 1 mM EDTA	soaking: 40–60 mM GTP and 25 mM Mg^{2+} and Ca^{2+}; In the presence of 2 mM $MnCl_2$
		pseudomonas phage φ6	1UVK	2.45	----	----	Mn^{2+}	Mg^{2+}	----	C: D454, E491, A495	----	elution from the column: 1 mM EDTA	soaking: 40–60 mM GTP and 25 mM Mg^{2+} and Ca^{2+}; In the presence of 2 mM $MnCl_2$
		pseudomonas phage φ6	1UVL	2.00	----	----	Mn^{2+}	----	----	C: D454, E491, A495	template	elution from the column: 1 mM EDTA	soaking: 40–60 mM GTP and 25 mM Mg^{2+} and Ca^{2+}; In the presence of 2 mM $MnCl_2$
		pseudomonas phage φ6	1UVN	3.00	Ca^{2+} (?)	Ca^{2+} (?)	Mn^{2+}	GTP	----	A: Y630, GTP<br>B: D453, V325, GTP<br>C: D454, E491, A495	polyribonucleotide	elution from the column: 1 mM EDTA	soaking: 40–60 mM GTP and 25 mM Mg^{2+} and Ca^{2+}; In the presence of 2 mM $MnCl_2$
		pseudomonas phage φ6	1UVM	2.00	----	----	Mn^{2+}	----	----	C: D454, E491, A495	template	elution from the column: 1 mM EDTA	soaking: 40–60 mM GTP and 25 mM Mg^{2+} and Ca^{2+}; In the presence of 2 mM $MnCl_2$
		pseudomonas phage φ6	1WAC	3.00	----	----	----	----	----	----	----	----	----
		pseudomonas phage φ6	2JLG	2.80	----	Mn^{2+}	----	GTP	----	B: D435, V325, D324, GTP	polydeoxyribonucleotide, E491Q	elution from the column: 1 mM EDTA	Soaking: 5 mM $MnCl_2$ to the crystallization drop, followed a few seconds later by the addition of 5 mM $MgCl_2$ and, immediately after, 25 mM GTP
		pseudomonas phage φ6	2JLF	3.20	----	----	Mn^{2+}	----	----	C: D454, E491, A495	E491Q	elution from the column: 1 mM EDTA	crystallization: 2 mM $MnCl_2$
		pseudomonas phage φ6	2JL9	3.20	----	----	----	----	----	----	E491Q	elution from the column: 1 mM EDTA	----

Reoviridae	*Orthoreovirus*	mammalian reovirus	1N1H	2.80	Mn^{2+}	Mn^{2+}	----	CH1,G7M,GDP, GH3	initiation	A: D585, D734, D735, CH1<br>B: I586, D585, D734, CH1	polyribonucleotide	purification: 1 mM EDTA	soaking: 1 mM 3′-deoxy rGTP, 1 mM 3′-deoxy rCTP and 1.5 mM Mn^{2+}
		mammalian reovirus	1N35	2.50	Mn^{2+}	Mn^{2+}	----	CH1	elongation	A: D585, D734, D735, CH1<br>B: I586, D585, D734, CH1	2 polyribonucleotides	purification: 1 mM EDTA	soaking: 1 mM rGTP,1 mM 3′-deoxy rCTP and 1.5 mM Mn^{2+}
		mammalian reovirus	1N38	2.80	Mn^{2+}	Mn^{2+}	----	CH1, U3H	elongation	A: D585, D734, D735, CH1<br>B: I586, D585, D734, CH1	polyribonucleotide	purification: 1 mM EDTA	soaking: 1.5 mM Mn^{2+}, 1 mM 3′-deoxy rUTP, 1 mM 3′-deoxy rCTP, 1 mM rGTP
		mammalian reovirus	1MUK	2.50	----	----	----	----	----	----	----	purification: 1 mM EDTA	----
		mammalian reovirus	1MWH	2.50	----	----	Mn^{2+}	GTG	----	C: D585, D735, E780	----	purification: 1 mM EDTA	----
	Rotavirus	simian rotavirus	2R7R	2.60	----	----	----	----	----	----	polyribonucleotide	----	----
		simian rotavirus	2R7S	3.24	----	----	----	PO_4	----	----	polyribonucleotide	----	----
		simian rotavirus	2R7T	3.00	----	----	----	----	----	----	polyribonucleotide	----	----
		simian rotavirus	2R7U	3.10	----	----	----	----	----	----	polyribonucleotide	----	----
		simian rotavirus	2R7V	2.60	----	----	----	----	----	----	polyribonucleotide	----	----
		simian rotavirus	2R7W	2.60	----	----	----	GTP	----	----	polyribonucleotide	----	----
		simian rotavirus	2R7X	2.80	----	----	----	GTP	----	----	polyribonucleotide	----	----
		simian rotavirus	2R7Q	2.90	----	----	----	----	----	----	----	----	----
		simian rotavirus	2R7O	3.35	----	----	----	----	----	----	----	----	----

[1] (?) Indicates that the ion is likely to be an catalytic ion, but not in the catalytic site.

[2]Abbreviations for the ligands

153	(2s)-2-[(2,4-dichloro-benzoyl)-(3-trifluoromethyl-benzyl)-amino]-3-phenyl-propionic acid
154	(2s)-2-[(5-benzofuran-2-yl-thiophen-2-ylmethyl)-(2,4-dichloro-benzoyl)-amino]-3-phenyl-propionic acid
221	(2r,3r)-3-{[3,5-bis(trifluoromethyl)phenyl]amino}-2-cyano-3-thioxopropanamide
321	n-{3-[5-hydroxy-2-(3-methylbutyl)-3-oxo-6- thiophen-2-yl-2,3-dihydropyridazin-4-yl]- 1,1-dioxido-2h-1,4-benzothiazin-7-yl}methanesulfonamide
452	2-{[n-(2-acetyl-5-chloro-4-fluorophenyl)glycyl]amino}benzoic acid
617	2-{[(4-chlorophenoxy)acetyl]amino}benzoic acid
698	(2s,4s,5r)-1-(4-tert-butylbenzoyl)-2-isobutyl-5-(1,3-thiazol-2-yl)pyrrolidine-2,4-dicarboxylic acid
699	(2s,4s,5r)-2-isobutyl-5-(2-thienyl)-1-[4-(trifluoromethyl)benzoyl]pyrrolidine-2,4-dicarboxylic acid
885	1-[(2-amino-4-chloro-5-methylphenyl)sulfonyl]-l-proline
888	3-(1,1-dioxido-4h-1,2,4-benzothiadiazin-3-yl)-4-hydroxy-1-(3-methylbutyl)quinolin-2(1h)-one
15P	polyethylene glycol (n=34)
1B8	(7r)-14-cyclohexyl-7-{[2-(dimethylamino)ethyl](methyl)amino}-7,8-dihydro-6h-indolo[1,2-e][1,5]benzoxazocine-11-carboxylic acid
1PD	2-({3-[5-hydroxy-2-(3-methylbutyl)-3-oxo- 6-(1,3-thiazol-5-yl)-2,3-dihydropyridazin- 4-yl]-1,1-dioxido-2h-1,2,4-benzothiadiazin- 7-yl}oxy)acetamide
2PD	5-hydroxy-4-(7-methoxy-1,1-dioxido-2h-1,2,4- benzothiadiazin-3-yl)-2-(3-methylbutyl)-6- phenylpyridazin-3(2h)-one
3MS	n-{3-[4-hydroxy-1-(3-methylbutyl)-2-oxo-1,2- dihydropyrrolo[1,2-b]pyridazin-3-yl]-1,1- dioxido-2h-1,2,4-benzothiadiazin-7-yl}methanesulfonamide
4MS	n-({3-[(5s)-5-tert-butyl-1-(3-chloro-4-fluorobenzyl)- 4-hydroxy-2-oxo-2,5-dihydro-1h-pyrrol-3-yl]- 1,1-dioxido-1,2-benzisothiazol-7-yl}methyl)methanesulfonamide
5EE	5r-(3,4-dichlorophenylmethyl)-3-(2-thiophenesulfonylamino)- 4-oxo-2-thionothiazolidine
5FU	5-fluoro-uridine-5'-monophosphate
5H	5r-(4-bromophenylmethyl)-3-(benzenesulfonylamino)- 4-oxo-2-thionothiazolidine
5X	5r-(2e-methyl-3-phenyl-allyl)-3-(benzenesulfonylamino)- 4-oxo-2-thionothiazolidine
77Z	2-({(3r)-3-[(3s)-1-(3-methylbutyl)-2,4-dioxo- 1,2,3,4-tetrahydroquinolin-3-yl]-1,1-dioxido- 3,4-dihydro-2h-1,2,4-benzothiadiazin-7-yl}oxy)acetamide
79Z	5-cyclopropyl-2-(4-fluorophenyl)-6-[(2-hydroxyethyl)(methylsulfonyl)amino]- n-methyl-1-benzofuran-3-carboxamide
ACT	acetate ion
ACY	acetic acid
AG0	(6r)-6-cyclopentyl-6-[2-(2,6-diethylpyridin- 4-yl)ethyl]-3-[(5,7-dimethyl[1,2,4]triazolo[1,5- a]pyrimidin-2-yl)methyl]-4-hydroxy-5,6-dihydro- 2h-pyran-2-one
AG6	n-[(benzyloxy)carbonyl]-l-alpha-glutamyl- n-[(1s)-4-oxo-4-phenyl-1-propylbut-2-en-1- yl]-l-phenylalaninamide
ATP	adenosine-5'-triphosphate
B34	(5s)-1-benzyl-3-(1,1-dioxido-1,2-benzisothiazol- 3-yl)-4-hydroxy-5-(1-methylethyl)-1,5-dihydro- 2h-pyrrol-2-one
B42	n-{3-[(5r)-1-cyclopentyl-4-hydroxy-5-methyl- 5-(3-methylbutyl)-2-oxo-1,2,5,6-tetrahydropyridin- 3-yl]-1,1-dioxido-4h-1,2,4-benzothiadiazin- 7-yl}methanesulfonamide
B5P	n-{3-[5-hydroxy-8-(3-methylbutyl)-7-oxo-7,8- dihydroimidazo[1,2-a]pyrimidin-6-yl]-1,1- dioxido-4h-1,2,4-benzothiadiazin-7-yl}methanesulfonamide
B80	n-{3-[(6s)-6-ethyl-1-(4-fluorobenzyl)-4-hydroxy- 2-oxo-1,2,5,6-tetrahydropyridin-3-yl]-1,1- dioxido-2h-1,2,4-benzothiadiazin-7-yl}methanesulfonamide
BIW	1-[2-(4-carboxypiperidin-1-yl)-2-oxoethyl]- 3-cyclohexyl-2-furan-3-yl-1h-indole-6-carboxylic acid
CCT	5-(4-cyanophenyl)-3-{[(2-methylphenyl)sulfonyl]amino}thiophene- 2-carboxylic acid
CH1	3'-deoxy-cytidine-5'-triphosphate
Cl^-	cloride ion
CMF	3-cyclohexyl-1-(2-morpholin-4-yl-2-oxoethyl)- 2-phenyl-1h-indole-6-carboxylic acid
CSG	2'-amino-2'-deoxycytidine 5'-(tetrahydrogen triphosphate)
CTP	cytidine-5'-triphosphate
DCP	2'-deoxycytidine-5'-triphosphate
DCT	2',3'-dideoxycytidine 5'-triphosphate
DEY	(2r)-2-({3-[5-hydroxy-2-(3-methylbutyl)-3- oxo-6-thiophen-2-yl-2,3-dihydropyridazin- 4-yl]-1,1-dioxido-2h-1,2,4-benzothiadiazin- 7-yl}oxy)propanamid
DMX	3-[benzyl(dimethyl)ammonio]propane-1-sulfonate
DPO	diphosphate
FIH	5-(4-fluorophenyl)-3-{[(4-methylphenyl)sulfonyl]amino}thiophene- 2-carboxylic acid
FUTP	fluoro-uridine-5'-triphosphate
G7M	n7-methyl-guanosine-5'-monophosphate
GDP	guanosine-5'-diphosphate
GH3	3'-deoxy-guanosine-5'-triphosphate

GOL	glycerol
GTG	7-methyl-guanosine-5'-triphosphate-5'-guanosine
GTP	guanosine-5'-triphosphate
H59	n-{3-[(5s)-5-(1,1-dimethylpropyl)-1-(4-fluoro- 3-methylbenzyl)-4-hydroxy-2-oxo-2,5-dihydro- 1h-pyrrol-3-yl]-1,1-dioxido-4h-1,4-benzothiazin- 7-yl}methanesulfonamide
H5S	(5s)-5-tert-butyl-1-(4-fluoro-3-methylbenzyl)- 4-hydroxy-3-[8-(methylsulfonyl)-1,1-dioxido- 6,7,8,9-tetrahydroisothiazolo[4,5-h]isoquinolin- 3-yl]-1,5-dihydro-2h-pyrrol-2-one
HJZ	3-[2-(trans-4-methylcyclohexyl)phenyl]-5-phenylthiophene-2-carboxylic acid
IPA	isopropyl alcohol
IPC	3-[isopropyl(4-methylbenzoyl)amino]-5-phenylthiophene- 2-carboxylic acid
IX6	11s)-10-[(2,5-dimethyl-1,3-oxazol-4-yl)carbonyl]-11-{2-fluoro-4-[(2-methylprop-2-en-1-yl)oxy]phenyl}-3,3-dimethyl-2,3,4,5,10,11-hexahydrothiopyrano[3,2-b][1,5]benzodiazepin-6-ol 1,1-dioxide
JPC	3-[(2,4-dichlorobenzoyl)(isopropyl)amino]- 5-phenylthiophene-2-carboxylic acid
JTP	n-[(13-cyclohexyl-6,7-dihydroindolo[1,2-d][1,4]benzoxazepin- 10-yl)carbonyl]-2-methyl-l-alanine
K^{+}	kalium ion
LT6	2-(2-{[(1s)-1-benzyl-2-hydroxyethyl]amino}- 2-oxoethoxy)-n-butyl-6-fluoro-n-methylbenzamide
Mg^{2+}	magnesium ion
Mn^{2+}	manganese ion
MSE	selenomethionine
N34	n-{3-[1-(3,3-dimethyl-butyl)-4-hydroxy-2- oxo-1,2,4a,5,6,7-hexahydro-pyrrolo[1,2-b]pyridazin- 3-yl]-1,1-dioxo-1,2-dihydro-1lambda6-benzo[1,2,4]thiadiazin- 7-yl}-methanesulfonamide
N35	n-{3-[5-hydroxy-2-(3-methylbutyl)-3-oxo-6- pyrrolidin-1-yl-2,3-dihydropyridazin-4-yl]- 1,1-dioxido-2h-1,2,4-benzothiadiazin-7-yl}methanesulfonamide
N3H	n-{3-[5-hydroxy-2-(3-methylbutyl)-3-oxo-6- thiophen-2-yl-2,3-dihydropyridazin-4-yl]- 1,1-dioxido-2h-1,2,4-benzothiadiazin-7-yl}methanesulfonamide
N5C	5-nitrocytidine 5'-(tetrahydrogen triphosphate)
Na^{+}	natrium ion
NH1	3-(4-amino-2-tert-butyl-5-methyl-phenylsulfanyl)-6-cyclopentyl-4-hydroxy-6-[2-(4-hydroxy-phenyl)-ethyl]-5,6-dihydro-pyran-2-one
Ni^{2+}	nickel ion
NN2	1-(2-cyclopropylethyl)-3-(1,1-dioxido-2h- 1,2,4-benzothiadiazin-3-yl)-6-fluoro-4-hydroxyquinolin- 2(1h)-one
NN3	3-{isopropyl[(trans-4-methylcyclohexyl)carbonyl]amino}- 5-phenylthiophene-2-carboxylic acid
PEG	di(hydroxyethyl)ether
PFI	(6s)-6-cyclopentyl-6-[2-(3-fluoro-4-isopropoxyphenyl)ethyl]- 4-hydroxy-5,6-dihydro-2h-pyran-2-one
PGE	triethylene glycol
PH7	(2z)-2-(benzoylamino)-3-[4-(2-bromophenoxy)phenyl]- 2-propenoic acid
PH9	(2z)-2-[(1-adamantylcarbonyl)amino]-3-[4- (2-bromophenoxy)phenyl]prop-2-enoic acid
PHQ	benzyl chlorocarbonate
PO4	phosphate ion
POO	3-cyclohexyl-1-(2-{methyl[(1-methylpiperidin-3-yl)methyl]amino}-2-oxoethyl)-2-phenyl-1h-indole-6-carboxylic acid
POP	pyrophosphate 2-
PPV	pyrophosphate
PXN	(2s)-1-[3-{[(2r)-2-hydroxypropyl]oxy}-2,2- bis({[(2r)-2-hydroxypropyl]oxy}methyl)propoxy]propan- 2-ol
QQ3	(3r)-3-(4-methyl-1,3-dioxo-1,3-dihydro-2h- pyrrolo[3,4-c]quinolin-2-yl)hexanoic acid
RTP	ribavirin triphosphate
SNH	5'-acetyl-4-{[(2,4-dimethylphenyl)sulfonyl]amino}- 2,2'-bithiophene-5-carboxylic acid
SO4	sulfate ion
SX1	2-amino-5-bromobenzoic acid
SX2	4-[(5-bromopyridin-2-yl)amino]-4-oxobutanoic acid
SX3	4-bromo-2-{[(3r,5s)-3,5-dimethylpiperidin- 1-yl]carbonyl}aniline
SX4	4-bromo-2-{[(2r)-2-(2-chlorobenzyl)pyrrolidin- 1-yl]carbonyl}aniline
SX5	4-[(4-bromo-2-{[(3r,5s)-3,5-dimethylpiperidin- 1-yl]carbonyl}phenyl)amino]-4-oxobutanoic acid
SX6	n-(4-bromo-2-{[(3r,5s)-3,5-dimethylpiperidin- 1-yl]carbonyl}phenyl)-4-morpholin-4-yl-4- oxobutanamide
T18	n-{3-[6-fluoro-1-(4-fluorobenzyl)-4-hydroxy- 2-oxo-1,2-dihydroquinolin-3-yl]-1,1-dioxido- 4h-1,4-benzothiazin-7-yl}methanesulfonamide
U3H	3'-deoxy-uridine 5'-triphosphate
U5P	uridine-5'-monophosphate
UTP	uridine 5'-triphosphate
VGC	4-(2-aminoethoxy)-n-(3-chloro-5-piperidin- 1-ylphenyl)-3,5-dimethylbenzamide
VGI	2-(3-bromophenyl)-6-[(2-hydroxyethyl)amino]-1h-benzo[de]isoquinoline-1,3(2h)-dione
VR1	(2s)-2-({(5z)-5-[(5-ethyl-2-furyl)methylene]- 4-oxo-4,5-dihydro-1,3-thiazol-2-yl}amino)- 2-(4-fluorophenyl)-n-[(4-nitrophenyl)sulfonyl]acetamide
VRX	(2s)-({(5z)-5-[(5-ethyl-2-furyl)methylene]- 4-oxo-4,5-dihydro-1,3-thiazol-2-yl}amino)(4- fluorophenyl)acetic acid
VXR	(5z)-5-[(5-ethyl-2-furyl)methylene]-2-{[(s)- (4-fluorophenyl)(1h-tetrazol-5-yl)methyl]amino}- 1,3-thiazol-4(5h)-one
XNC	(11s)-11-[4-(benzyloxy)-2-fluorophenyl]-3,3- dimethyl-10-[(6-methylpyridin-2-yl)carbonyl]- 2,3,4,5,10,11-hexahydrothiopyrano[3,2-b][1,5]benzodiazepin- 6-ol 1,1-dioxide
XND	(11r)-10-acetyl-11-(2,4-dichlorophenyl)-6- hydroxy-3,3-dimethyl-2,3,4,5,10,11-hexahydro- 1h-dibenzo[b,e][1,4]diazepin-1-one
XNI	(11s)-10-acetyl-11-[4-(benzyloxy)-3-chlorophenyl]- 3,3-dimethyl-2,3,4,5,10,11-hexahydro-1h-dibenzo[b,e][1,4]diazepin- 1-one
XNZ	(11r)-10-acetyl-11-[4-(benzyloxy)-2-chlorophenyl]- 6-hydroxy-3,3-dimethyl-2,3,4,5,10,11-hexahydro- 1h-dibenzo[b,e][1,4]diazepin-1-one
YAK	n-{3-[(4ar,7as)-1-(4-fluorobenzyl)-4-hydroxy- 2-oxo-2,4a,5,6,7,7a-hexahydro-1h-cyclopenta[b]pyridin- 3-yl]-1,1-dioxido-2h-1,2,4-benzothiadiazin- 7-yl}methanesulfonamide
Zn^{2+}	zinc ion
