# Supplementary material for: Evidence for a Non-Catalytic Ion-Binding Site in Multiple RNA-Dependent RNA Polymerases
Source: PLoS One. 2012 Jul 11;7(7):e40581. doi: 10.1371/journal.pone.0040581 (PMC3394715; doi:10.1371/journal.pone.0040581)
Supplement: Table S2 — The RdRp protein sequences used in the multiple alignment. The family name, species and the NCBI access number are given. (PDF) [file pone.0040581.s003.pdf]

Supporting information Table S2 The RdRp protein sequences used in the multiple alignments

Family/Subfamily	Species	The access numbers of NCBI protein database
Leviviridae	enterobacteria phage BZ13	GI: 254547574
Leviviridae	enterobacteria phage MS2	GI: 146150142
Leviviridae	enterobacteria phage FI	GI: 254547619
Leviviridae	enterobacteria phage Qβ	GI: 9630321
Cystoviridae	pseudomonas phage φ6	GI: 94449926
Cystoviridae	pseudomonas phage φ8	GI: 7532971
Cystoviridae	pseudomonas phage φ12	GI: 22855213
Cystoviridae	pseudomonas phage φ13	GI: 22855206
Sedoreovirinae	eriocheir sinensis reovirus	GI: 47155947
Sedoreovirinae	micromonas pusilla reovirus	GI: 123811909
Sedoreovirinae	bluetongue virus	GI: 297655564
Sedoreovirinae	rice dwarf virus	GI: 222499
Sedoreovirinae	rotavirus A	GI: 156617974
Sedoreovirinae	banna virus	GI: 82005806
Spinareovirinae	aquareovirus C	GI: 38147303
Spinareovirinae	Colorado tick fever virus	GI: 81968439
Spinareovirinae	cypovirus 1	GI: 14993634
Spinareovirinae	aedes pseudoscutellaris reovirus	GI: 123840414
Spinareovirinae	Fiji disease virus	GI: 81952235
Spinareovirinae	mammalian orthoreovirus	GI: 17154964
Spinareovirinae	mycoreovirus 1	GI: 189022225
Spinareovirinae	rice ragged stunt virus	GI: 3641393
