# Supplementary material for: Evidence for a Non-Catalytic Ion-Binding Site in Multiple RNA-Dependent RNA Polymerases
Source: PLoS One. 2012 Jul 11;7(7):e40581. doi: 10.1371/journal.pone.0040581 (PMC3394715; doi:10.1371/journal.pone.0040581)
Supplement: Table S3 — The reverse transcriptase and DNA-dependent polymerase structures. The PDBids of the analyzed reverse transcriptses and DNA-dependent polymerases are given. In addition, species, resolution, ligands, mutations, observed ions close to the catalytic site and their coordinating amino acids are provided for each structure. (PDF) [file pone.0040581.s004.pdf]

Supporting information Table S3. The reverse transcriptase and DNA-dependent polymerase structures

Polymerase family/type	Polymerase	Species	PDB ID	Resolution (Å)	Catalytic ion A[1]	Catalytic ion B	Non-catalytic ion, C	Ions at ~6 Å distance from the catalytic site	Amino acids coordinating catalytic or non-catalytic ions	Other ligands[2]	Mutations
reverse transcriptase	reverse transcriptase	human immunodeficiency virus	1N6Q	3.00	----	----	Mg^{2+}	----	C: D186, D110	MRG, ATM, template, primer (modified ATM), antibody	C280S, Q258C
	reverse transcriptase	human immunodeficiency virus	3KLE	3.20	Mg^{2+} (?)	----	----	----	A: D185, V111, D110, ZP4	GOL, SWE, ZP4, template, primer	M41L, D67N, K70R, T215Y, K219Q, Q258C, C280S
	reverse transcriptase	human immunodeficiency virus	3KLF	3.15	Mg^{2+} (?)	----	----	----	A: D185, V111, D110, ZP4	GOL, ZP4, template, primer	C280S, Q258C
	reverse transcriptase	human immunodeficiency virus	3KK1	2.70	----	Mg^{2+}	----	----	B: D185, V111, D110, 914	914, SO_4, template, primer (modified DOC)	C280S, Q258C
	reverse transcriptase	human immunodeficiency virus	3KK2	2.90	----	Mg^{2+}	----	----	B: D185, V111, D110, DTP	SO_4, template, primer (modified DOC	----
	reverse transcriptase	human immunodeficiency virus	2IAJ	2.50	Mn^{2+}	Mn^{2+}	----	----	A: D185, D186, D110, ATP<br>B: D185, V111, D110, ATP	ATP, GOL, Na^+	K103N, Y181C, C280S
Family A	DNA polymerase I	*Bacillus stearothermophilus*	2BDP	1.80	Mg^{2+}	----	----	----	A: D653,Y654, D830	SO_4, template, primer	----
	DNA polymerase I	*Bacillus stearothermophilus*	4BDP	1.80	Mg^{2+}	----	----	----	A: D653, D830, Y654	----	----
	DNA polymerase I	*Escherichia coli*	1U45	2.01	Mg^{2+}	----	----	----	A: D653, Y654, D830	SO_4, SUC, template (modifies 8OG), primer	----
	DNA polymerase I	*Escherichia coli*	1U47	2.00	Mg^{2+}	----	----	----	A: D653, Y654, D830	SO_4, SUC, template (modifies 8OG), primer	----
	DNA polymerase I	*Escherichia coli*	1U48	2.10	Mg^{2+}	----	----	----	A: D653, Y654, D830	SO_4, SUC, template (modifies 8OG), primer	----
	DNA polymerase I	*Escherichia coli*	1U49	2.15	Mg^{2+}	----	----	----	A: D653, Y654, D830	SO_4, SUC, template, primer	----
	DNA polymerase I	*Escherichia coli*	1U4B	1.60	Mg^{2+}	----	----	----	A: D653, Y654, D830	SO_4, SUC	----
	DNA polymerase I	*Geobacillus stearothermophilus*	1L3S	1.70	Mg^{2+}	----	----	----	A: D653, Y654, D830	SO_4, SUC, template, primer	----
	DNA polymerase I	*Geobacillus stearothermophilus*	1L3T	1.70	Mg^{2+}	----	----	----	A: D653, Y654, D830	SO_4, SUC, template, primer	----
	DNA polymerase I	*Geobacillus stearothermophilus*	1L3U	1.80	Mg^{2+}	----	----	----	A: D653, Y654, D830	SO_4, SUC, template, primer	----
	DNA polymerase I	*Geobacillus stearothermophilus*	1L3V	1.71	Mg^{2+}	----	----	----	A: D653, Y654, D830	SO_4, SUC, template, primer	----
	DNA polymerase I	*Geobacillus stearothermophilus*	1L5U	1.95	Mg^{2+}	----	----	----	A: D653, Y654, D830	SO_4, SUC, template, primer	----
	DNA polymerase I	*Geobacillus stearothermophilus*	1LV5	1.95	Mg^{2+}	Mn^{2+}	----	----	A: D653, D830, DCP<br>B: D653, Y654, D830, DCP	DCP, SO_4, primer, template	----
	DNA polymerase I	*Geobacillus stearothermophilus*	1NJX	1.65	Mg^{2+}	----	----	----	A: D653, Y654, D830	SO_4, SUC	----
	DNA polymerase I	*Geobacillus stearothermophilus*	1NJY	2.00	Mg^{2+}	----	----	----	A: D653, Y654, D830	SO_4, SUC, TTP	----
	DNA polymerase I	*Geobacillus stearothermophilus*	1NJZ	2.00	Mg^{2+}	----	----	----	A: D653, Y654, D830	SO_4, SUC, template, primer	----
	DNA polymerase I	*Geobacillus stearothermophilus*	1NK4	1.60	Mg^{2+}	----	----	----	A: D653, Y654, D830	SO_4, SUC, template, primer	----
	DNA polymerase I	*Geobacillus stearothermophilus*	1NK5	2.10	Mg^{2+}	----	----	----	A: D653, Y654, D830	SO_4, SUC, template, primer	----

	DNA polymerase I	*Geobacillus stearothermophilus*	1NK7	1.90	Mg^{2+}	----	----	----	A: D653, Y654, D830	SO_4, SUC, template, primer	----
	DNA polymerase I	*Geobacillus stearothermophilus*	1NK8	1.90	Mg^{2+}	----	----	----	A: D653, Y654, D830	SO_4, SUC, template, primer	----
	DNA polymerase I	*Geobacillus stearothermophilus*	1NK9	1.90	Mg^{2+}	----	----	----	A: D653, Y654, D830	SO_4, SUC, template, primer	----
	DNA polymerase I	*Geobacillus stearothermophilus*	1NKB	2.00	Mg^{2+}	----	----	----	A: D653, Y654, D830	SO_4, SUC, template, primer	----
	DNA polymerase I	*Geobacillus stearothermophilus*	1NKC	1.80	Mg^{2+}	----	----	----	A: D653, Y654, D830	SO_4, SUC, template, primer	----
	DNA polymerase I	*Geobacillus stearothermophilus*	1NKE	1.80	Mg^{2+}	----	----	----	A: D653, Y654, D830	SO_4, SUC, template, primer	----
	DNA polymerase I	*Geobacillus stearothermophilus*	1XC9	1.90	----	Mg^{2+}	----	----	B: D653, Y654	BAP, SO_4, SUC	----
	DNA polymerase I	*Geobacillus stearothermophilus*	2HHQ	1.80	Mg^{2+}	----	----	----	A: D830, Y654, D653	SO_4, SUC, primer, template	----
	DNA polymerase I	*Geobacillus stearothermophilus*	2HHS	1.80	Mg^{2+}	----	----	----	A: D830, Y654, D653	SO_4, SUC, primer, template	----
	DNA polymerase I	*Geobacillus stearothermophilus*	2HHT	2.05	Mg^{2+}	----	----	----	A: D830, Y654, D653	SO_4, SUC, primer, template	----
	DNA polymerase I	*stearothermophilus*	2HHU	1.80	Mg^{2+}	----	----	----	A: D830, Y654, D653	DCP. SO_4, SUC primer, template	----
	DNA polymerase I	*Geobacillus stearothermophilus*	2HHV	1.55	Mg^{2+}	----	----	----	A: D830, Y654, D653	SO_4, SUC, template, primer	----
	DNA polymerase I	*Geobacillus stearothermophilus*	2HHW	1.88	Mn^{2+}	----	----	----	A: D830, Y654, D653, DCT	23T, SO_4, SUC, template, primer (modified DDG)	D598A, F710Y
	DNA polymerase I	*Geobacillus stearothermophilus*	2HW3	1.98	Mg^{2+}	----	----	----	A: D653, Y654, D830	SO_4, SUC, template, primer	----
	DNA polymerase I	*Geobacillus stearothermophilus*	2HVH	2.49	Mn^{2+}	----	----	----	A: D830, Y654, D653, DCT	DCT, SO_4, SUC, template, primer (modified DDG)	----
	DNA polymerase I	*Geobacillus stearothermophilus*	2HVI	1.98	Mg^{2+}	Mg^{2+}	----	----	B: D653, Y654, D830, DCT	DCT, SO_4, SUC, template, primer (modified DDG)	----
	DNA polymerase I	*Thermus aquaticus*	1QSS	2.30	Mg^{2+}	Mg^{2+}	----	----	A: D610, D785, DG3 B: D610, D785, Y 611, DG3	DG3, template, primer (modified DDG)	A348E
	DNA polymerase I	*Thermus aquaticus*	1QSY	2.30	Mg^{2+}	Mg^{2+}	----	----	A: D610, D785, DDS B: D610, D785, Y 611, DDS	DDS, template, primer (modified 2DA)	A348E
	DNA polymerase I	*Thermus aquaticus*	1QTM	2.30	Mn^{2+}	Mn^{2+}	----	----	A: D610, D785, TTP B: D610, D785, Y 611, TTP	TTP, template, primer (modified 2DT)	----
	DNA polymerase I	*Thermus aquaticus*	2KTQ	2.30	Mg^{2+}	----	----	----	A: Y611, D785	----	----
	DNA polymerase I	*Thermus aquaticus*	3KTQ	2.30	Mg^{2+}	Mg^{2+}	----	----	A: D610, D785, DCT B: D610, D785, Y 611, DCT	DCT, template, primer (modified DOC)	----
	DNA polymerase I	*Thermus aquaticus*	3M8R	2.00	Mg^{2+}	Mg^{2+}	----	----	A: D785, D610, HXZ B: D785, D610, Y611, HXZ	HXZ, 15P, ACT, GOL, template, primer	----
	DNA polymerase I	*Thermus aquaticus*	3M8S	2.20	Mg^{2+}	Mg^{2+}	----	----	A: D610, D785, HXB B: D785, Y611, D610, HXB	HXB, 15P, ACT, GOL, PEG, template, primer (modified DOC)	----
	DNA polymerase I	*Thermus aquaticus*	3OJS	1.90	Mg^{2+}	Mg^{2+}	----	----	A: D785, D610, XJS B: D785, D610, Y611, XJS	XJS, ACT, GOL, PGE, primer (modified DOC), template	----
	DNA polymerase I	*Thermus aquaticus*	3OJU	2.00	Mg^{2+}	Mg^{2+}	----	----	A: D785, D610, SSJ B: D785, Y611, D610, SSJ	SSJ, GOL, PGE, primer, template	----
	DNA polymerase	enterobacteria phage T7	1T8E	2.54	Mg^{2+}	Mg^{2+}	----	----	A: D654, D475, DCT B: D654, A476, D475, DCT	DCT, primer (modified 2DT), template, thioredoxin, MES, PG4, SO_4	----
	DNA polymerase	enterobacteria phage T7	1TK0	2.30	Mg^{2+}	Mg^{2+}	----	----	A: D654, D475, DCT B: D654, A476, D475, DCT	DCT, primer (modified DDG), template, 1PE, MES, SO_4	----

	DNA polymerase	enterobacteria phage T7	1TK8	2.50	Mg^{2+}	Mg^{2+}	----	----	A: D654, D475, D3T<br>B: D654, A476, D475, D3T	D3T, primer (modified 2DA), template, MES, SO_4, 1PE	----
	DNA polymerase	enterobacteria phage T7	1TKD	2.49	Mg^{2+}	Mg^{2+}	----	----	A: D654, D475, D3T<br>B: D654, A476, D475, D3T	D3T, primer (modified DOC), template, MES, SO_4, 1PE	----
	DNA polymerase	enterobacteria phage T7	1SKR	2.40	Mg^{2+}	Mg^{2+}	----	----	A: D654, D475, DAD<br>B: D654, A476, D475 DAD	DAD, primer (modified 2DA), template	----
	DNA polymerase	enterobacteria phage T7	1SL2	2.30	Mg^{2+}	Mg^{2+}	----	----	A: D654, D475, DAD<br>B: D654, A476, D475, DAD	DAD, primer (modified 2DA), template	----
	DNA polymerase	enterobacteria phage T7	1T7P	2.20	Mg^{2+}	Mg^{2+}	----	----	A: D654, D475, DG3<br>B: D654, A475, D475, DG3	DG3, primer (modified 2DA), template	----
	DNA polymerase	enterobacteria phage T7	1ZYQ	2.70	Mg^{2+}	Mg^{2+}	----	----	A: D475, D654, DAD<br>B: D475, D476, D654, DAD	DAD, primer (modified DDG), template	----
Family B	DNA polymerase	bacillus phage φ29	2PYJ	2.03	Mg^{2+}	Mn^{2+}	----	----	A: D249, D458, DGT<br>B: D458, V250, D249, DGT	DGT, polydeoxyribonucleotide (modified DOC), polydeoxyribonucleotide	D12A, D66A
	DNA polymerase	bacillus phage φ29	2PYL	2.20	Mg^{2+}	Mg^{2+}	----	----	A: D458, 249, TTP<br>B: D249, V250, D458, TTP	TTP, polydeoxyribonucleotide	----
	DNA polymerase	escherichia phage RB69	1IG9	2.60	Ca^{2+}	Ca^{2+}	---	Ca^{2+}	A: D623, S624, D411, TTP<br>B: D623, L412, D411, TTP<br>(C: E686, D684)	TTP, primer (modified DOC), template	----
	DNA polymerase	escherichia phage RB69	2OZM	2.86	----	Mg^{2+}	----		B: D411, L412, D623, N5P	N5P, template, primer	D222A, D327A
	DNA polymerase	escherichia phage RB69	2OZS	2.75	----	Mg^{2+}	----		B: D411, L412, D623, DTP	DTP, template, primer	D222A, D327A
	DNA polymerase	escherichia phage RB69	3CQ8	2.50	Ca^{2+}	Ca^{2+}	----	Ca^{2+}	A: D623, D411, TTP<br>B: D623, L412, D411, TTP<br>(C: E686, E716)	TTP, template, primer, GOL, Na^+	L415F, D222A, D327A
	DNA polymerase	escherichia phage RB69	3KD5	2.69	Mg^{2+}	Mg^{2+}	----	----	A: D623, D411, PPF<br>B: D623, L412, D411, PPF	PPF, template, primer (modified 4DG)	D222A, V478W, N480S, I557M, N558A, R559L, L561V, I562T, I563C
	DNA polymerase	escherichia phage RB69	3LDS	3.00	Mn^{2+}	Mn^{2+}	----	----	A: D623, D411, DTP<br>B: D623, L412, D411, DTP	DTP, template, primer, SO_4	D222A, D327A, L561A, I563S
	DNA polymerase	escherichia phage RB69	3LZI	2.30	Ca^{2+}	Ca^{2+}	----	Ca^{2+}	A: D623, D411, DTP<br>B: D623, L412, D411<br>(C: E716)	DTP, template, primer	D222A, D327A, Y567A
	DNA polymerase	escherichia phage RB69	3LZJ	2.05	Ca^{2+}	Ca^{2+}	----	----	A: D623, D411, CTP<br>B: D623, L412, D411, CTP	CTP, template, primer	D222A, D327A, Y567A
	DNA polymerase	escherichia phage RB69	3NAE	2.00	Ca^{2+}	Ca^{2+}	----	----	A: D623, D411, DTP<br>B: D623, L412, D411, DTP	DTP, template, primer (modified DOC), non-template DNA	D222A, D327A, Y567A
	DNA polymerase	escherichia phage RB69	3NCI	1.79	Ca^{2+}	Ca^{2+}	----	----	A: D623, D411, DCP<br>B: D623, L412, D411, DCP	DCP, template, primer	D222A, D327A
	DNA polymerase	escherichia phage RB69	3NDK	2.00	Ca^{2+}	Ca^{2+}	----	Ca^{2+}	A: D623, D411, DCP<br>B: D623, L412, D611, DCP<br>(C: D411, E686, E716)	DCP, template, primer,	D222A, D327, Y567A
	DNA polymerase	escherichia phage RB69	3NE6	2.00	Ca^{2+}	Ca^{2+}	----	Ca^{2+}	A: D623, D411, DCP<br>B: D623, L412, D611, DCP<br>(C: E716)	DCP, template, primer	D222A, D327A, S565G, Y567A
	DNA polymerase	escherichia phage RB69	3NGI	1.89	Ca^{2+}	Ca^{2+}	----	Ca^{2+}	A: D623, D411, TTP<br>B: D623, L412, D411, TTP<br>(C: E686, E716 / E716)	TTP, template, primer	D222A, D327A, Y567A

	DNA polymerase	escherichia phage RB69	3NHG	2.50	Ca^{2+}	Ca^{2+}	----	Ca^{2+}	A: D623, D411, TTP<br>B: D623, L412, D411, TTP<br>(C: D411, TTP)	TTP, template, primer	D222A, D327A, S565G, Y567A
	DNA polymerase	escherichia phage RB69	3CFP	2.50	Ca^{2+}	Ca^{2+}	----	Ca^{2+}	A: D623, D411, TTP<br>B: D623, L412, D411, TTP<br>(C: E716)	TTP, template, primer (modified DOC)	----
	DNA polymerase	escherichia phage RB69	3CFR	2.40	Ca^{2+}	Ca^{2+}	----	Ca^{2+}	A: D623, S624, D411, TTP<br>B: D623, L412, D411, TTP<br>(C: D411, E686)	TTP, template, primer (modified DOC)	----
	DNA polymerase II	*Saccharomycetes cervisiae*	3IAY	2.00	Ca^{2+}	Ca^{2+}	----	Ca^{2+}	A: D764, DCP<br>B: D764, F609, D606, DCP<br>(C: D608, E802)	DCP, primer, (modified DOC), template, ACT	----
	DNA polymerase II	*Escherichia coli*	3K57	2.08	Mg^{2+}	Mg^{2+}	----	----	A: D547, D419, DTP<br>B: D547, Y420, D419, DTP	DTP, primer (modified DOC), template	D335N
	DNA polymerase II	*Escherichia coli*	3K58	2.05	Mg^{2+}	Mg^{2+}	----	----	A: D547, D419, TTP<br>B: D547, Y420, D419, TTP	TTP, primer (modified DOC), template	D335N
	DNA polymerase II	*Escherichia coli*	3K59	1.92	Mg^{2+}	Mg^{2+}	----	----	A: D547, D419, DCP<br>B: D547, D419, Y420, DCP	DCP, primer (modified DOC), template	D335N
	DNA polymerase II	*Escherichia coli*	3K5L	2.70	Mg^{2+}	Mg^{2+}	----	----	A: D547, D419, DTP<br>B: D547, Y420, D419, DTP	DTP, primer (modified DOC), template	D335N
	DNA polymerase II	*Escherichia coli*	3K5M	2.04	Ca^{2+}	Ca^{2+}	----	----	A: D547, D419, DG3<br>B: D547, Y420, D419, DG3	DG3, primer, template	D335N
	DNA polymerase II	*Escherichia coli*	3MAQ	2.40	Mg^{2+}	Mg^{2+}	----	----	A: D547, D419, DGT<br>B: D547, Y420, D419, DGT	DGT, primer (modified DOC), template	D335N
Family Y	DNA polymerase κ	*Homo sapiens*	2OH2	3.05	----	Mg^{2+}	----	----	A: D198, M108, D107, TTP	TTP, polydeoxyribonucleotides	----
	DNA polymerase κ	*Homo sapiens*	2W7O	3.16	Ca^{2+}	Ca^{2+}	----	----	A: D107, M108, D198, DGT<br>B: D107, D325, DGT	DGT, primer, template	L36S
	DNA polymerase κ	*Homo sapiens*	2W7P	3.71	Ca^{2+}	----	----	----	A: D107, M108, D198, DTP	DTP, primer, template	L36S
	DNA polymerase κ	*Homo sapiens*	3IN5	3.20	----	Mg^{2+}	----	----	A: D198, M108, D107, ATP	ATP	C543A
	DNA polymerase IV	*Sulfolobus acidocaldarius*	3BQ1	2.70	----	Ca^{2+}	----	----	B: D105, F8, D7, DG3	DG3, primer, template	----
	DNA polymerase IV	*Sulfolobus acidocaldarius*	3BQ2	2.70	Ca^{2+}	----	----	----	A: D105	primer, template	----
	DNA polymerase IV	*Sulfolobus solfataricus*	1JX4	1.70	----	Ca^{2+}	----	----	B: D105, F8,D7, ADI	ADI, primer, template	----
	DNA polymerase IV	*Sulfolobus solfataricus*	1JXL	2.10	Mg^{2+}	Ca^{2+}	----	----	A: D105, E106, D7, DG3<br>B: D105, F8, D7, DG3	DG3, EDO, primer, template	----
	DNA polymerase IV	*Sulfolobus solfataricus*	1N48	2.20	Ca^{2+}	Ca^{2+}	----	----	A: D105, E106, D7, ATP<br>B: D105, F8, D7, ATP	ATP, primer, template	----
	DNA polymerase IV	*Sulfolobus solfataricus*	1N56	2.40	Mg^{2+}	Mg^{2+}	----	----	A: E106, D7, ATP<br>B: D105, D7, F8	ATP, primer, template	----
	DNA polymerase IV	*Sulfolobus solfataricus*	1RYR	2.28	Ca^{2+}	Ca^{2+}	----	----	A: D105, ATP<br>B: D105, F8, D7, ATP	ATP, primer, template	----
	DNA polymerase IV	*Sulfolobus solfataricus*	1RYS	2.03	Ca^{2+}	Ca^{2+}	----	----	A: D105, ATP<br>B: D105, D7, F8, ATP	ATP, EDO, primer, template	----
	DNA polymerase IV	*Sulfolobus solfataricus*	1S0M	2.70	Mg^{2+}	Ca^{2+}	----	----	A: D105, E106, D7, DTP<br>B: D105, D7, F8, DTP	DTP, BAP, primer, template	----
	DNA polymerase IV	*Sulfolobus solfataricus*	1S0N	2.80	Ca^{2+}	Ca^{2+}	----	----	A: D7, E106, DCP<br>B: D7 D105, F8, DCP	DCP, primer, template	----
	DNA polymerase IV	*Sulfolobus solfataricus*	1S0O	2.10	Ca^{2+}	Ca^{2+}	----	----	A: D105, E106, D7, TTP<br>B: D105, F8, D7, TTP	TTP, primer, template	----
	DNA polymerase IV	*Sulfolobus solfataricus*	1S10	2.10	Ca^{2+}	Ca^{2+}	----	----	A: D105, E106, D7, DCP<br>B: D105, F8,D7, DCP	DCP, primer, template	----

	DNA polymerase IV	*Sulfolobus solfataricus*	1S97	2.40	Ca^{2+}	----	----	----	A: D105, F8, D7, DCT	DCT, primer template	----
	DNA polymerase IV	*Sulfolobus solfataricus*	1S9F	2.00	Ca^{2+}	----	----	----	A: D105, F8, D7, DDY	DDY, Mg^{2+}, primer, template	----
	DNA polymerase IV	*Sulfolobus solfataricus*	2AGO	2.85	Mg^{2+}	Ca^{2+}	----	----	A: D105, E106, POP<br>B: D105, F8, D7, POP	POP, primer, template	----
	DNA polymerase IV	*Sulfolobus solfataricus*	2AGP	2.90	Mg^{2+}	Ca^{2+}	----	----	A: E106, D105, DGT<br>B: D105, D7, F8, DGT	DGT, primer (modified DOC),<br>template	----
	DNA polymerase IV	*Sulfolobus solfataricus*	2AGQ	2.10	Mg^{2+}	Ca^{2+}	----	----	A: E106, D7<br>B: D105, D7, F8, DTP	DTP, primer (modified DOC),<br>template	----
	DNA polymerase IV	*Sulfolobus solfataricus*	2ASD	1.95	Ca^{2+}	Ca^{2+}	----	----	A: E106, D7<br>B: D105, D7, F8, DCP	DCP, primer (modified DDG),<br>template	----
	DNA polymerase IV	*Sulfolobus solfataricus*	2ASJ	2.35	Ca^{2+}	----	----	----	A: D105, E106, D7	primer (modified DDG), template	----
	DNA polymerase IV	*Sulfolobus solfataricus*	2ASL	2.56	Ca^{2+}	----	----	----	A: D105, D7	primer (modified DOC), template	----
	DNA polymerase IV	*Sulfolobus solfataricus*	2BQ3	2.00	Ca^{2+}	Ca^{2+}	----	----	A: E106, D7<br>B: D105, F8	primer, template	----
	DNA polymerase IV	*Sulfolobus solfataricus*	2BQR	2.37	Ca^{2+}	Ca^{2+}	----	----	A: E106, D105, D7, DTP<br>B.D105, F8, D7, DTP	DTP, primer, template	----
	DNA polymerase IV	*Sulfolobus solfataricus*	2BQU	2.50	Ca^{2+}	Ca^{2+}	----	----	A: E106, D7, DAD<br>B: D105, F8, D7, DAD	DAD, primer, template	----
	DNA polymerase IV	*Sulfolobus solfataricus*	2BR0	2.17	Ca^{2+}	Ca^{2+}	----	----	A: E106, D105, D7<br>B: D105, F8, D7	DG, primer, template	----
	DNA polymerase IV	*Sulfolobus solfataricus*	2C22	2.56	Ca^{2+}	Ca^{2+}	----	----	A: E106, D105, D7, DGT<br>B:105, F8, D7, DGT	DGT, primer, template	----
	DNA polymerase IV	*Sulfolobus solfataricus*	2C28	2.27	Ca^{2+}	Ca^{2+}	----	----	A: D7, D105, E106, DG<br>B: D105, F8, D7	DG, primer, template	----
	DNA polymerase IV	*Sulfolobus solfataricus*	2C2D	2.57	Ca^{2+}	Ca^{2+}	----	----	A: D7, D105, E106, DTP<br>B: D7, F8, D105, DTP	DTP, primer, template	----
	DNA polymerase IV	*Sulfolobus solfataricus*	2C2E	2.61	Ca^{2+}	Ca^{2+}	----	----	A: D105, E106, D7, DCT<br>B: D105, F8, D7, DCT	DCT, primer, template	----
	DNA polymerase IV	*Sulfolobus solfataricus*	2C2R	2.55	Ca^{2+}	Ca^{2+}	----	----	A: D105, E106, D7, DCT<br>B: D105, F8, D7, DCT	DCT, primer, template	----
	DNA polymerase IV	*Sulfolobus solfataricus*	2IA6	2.50	----	Ca^{2+}	----	----	B: D105, F8, D7, ATP	ATP, BAP, EDO, GOL, PO_4, primer,<br>template	----
	DNA polymerase IV	*Sulfolobus solfataricus*	2IBK	2.25	Ca^{2+}	Ca^{2+}	----	----	A: D105. D106, D7, POP<br>B: D105, F8, D7, POP	POP, BAP, EDO, GOL, primer,<br>template	----
	DNA polymerase IV	*Sulfolobus solfataricus*	2IMW	2.05	----	Ca^{2+}	----	----	B: D105, F8, D7, DDS	DDS, EDO, primer, template	----
	DNA polymerase IV	*Sulfolobus solfataricus*	2J6S	2.50	Ca^{2+}	Ca^{2+}	----	----	A: D105, E106, D7. DTP<br>B: D105, F8, D7, DTP	DTP, primer, template	----
	DNA polymerase IV	*Sulfolobus solfataricus*	2J6T	2.60	Ca^{2+}	Ca^{2+}	----	----	A: D105, E106, D7, DTP<br>B: D105, F8, D7, DTP	DTP, primer , template	----
	DNA polymerase IV	*Sulfolobus solfataricus*	2J6U	2.50	Ca^{2+}	Ca^{2+}	----	----	A: D108, E109, D10, DGT<br>B: D108, D10, F11, DGT	DGT, primer, template	----
	DNA polymerase IV	*Sulfolobus solfataricus*	2JEF	2.17	Ca^{2+}	Ca^{2+}	----	----	A: D105, E106, D7, DGT<br>B: D105, F8, D7, DGT	DGT, primer, (modified DOC),<br>template	----
	DNA polymerase IV	*Sulfolobus solfataricus*	2JEG	2.38	Ca^{2+}	Ca^{2+}	----	----	A: D105, E106, D7, DGT<br>B: D105, F8, D7, K159	DGT, primer, (modified DOC),<br>template	----
	DNA polymerase IV	*Sulfolobus solfataricus*	2JEI	2.39	Ca^{2+}	Ca^{2+}	----	----	A: D105, E106, D7, DGT<br>B: D105, F8, D7, DGT	DGT, primer, template	----
	DNA polymerase IV	*Sulfolobus solfataricus*	2JEJ	1.86	Ca^{2+}	Ca^{2+}	----	----	A: D105, E106, D7, DGT<br>B: D105, F8, D7, DGT	DGT, primer, template	----
	DNA polymerase IV	*Sulfolobus solfataricus*	2RDJ	2.20	----	Ca^{2+}	----	----	B: D105, F8, D7, TMP	TMP, GOL, primer, template	----
	DNA polymerase IV	*Sulfolobus solfataricus*	2V4Q	2.60	Ca^{2+}	Ca^{2+}	----	----	A: D105, E106, D7, DGT<br>B: D105, F8, D7, DGT	DGT, primer, template	----

	DNA polymerase IV	*Sulfolobus solfataricus*	2V4R	2.50	Ca^{2+}	Ca^{2+}	----	----	A: D106, E107, D6, DGT<br>B: D106, F9, D8, DGT	DGT, primer, template	----
	DNA polymerase IV	*Sulfolobus solfataricus*	2W8K	3.10	Mg^{2+}	Mg^{2+}	----	----	A: E106, D7, DGT<br>B: D105, F8, D7, DGT	DGT, primer, (modified DOC),<br>template	----
	DNA polymerase IV	*Sulfolobus solfataricus*	2W8L	3.00	Mg^{2+}	Mg^{2+}	----	----	A: D105, E106, D7, DGT<br>B: D105, F8, D7, K159	DGT, primer, (modified DOC),<br>template	----
	DNA polymerase IV	*Sulfolobus solfataricus*	2W9A	2.60	Mg^{2+}	Mg^{2+}	----	----	A: D105, E106, D7, DGT<br>B: D105, F8, D7, DGT	DGT, primer, (modified DOC),<br>template	----
	DNA polymerase IV	*Sulfolobus solfataricus*	2W9B	2.28	----	Mg^{2+}	----	----	B: D105, F8, D7	primer, (modified DOC), template	----
	DNA polymerase IV	*Sulfolobus solfataricus*	2W9C	2.90	Mg^{2+}	Mg^{2+}	----	----	A: D105, E106, TTP<br>B: D105, F8, D7, TTP	TTP, primer, (modified DOC),<br>template	----
	DNA polymerase IV	*Sulfolobus solfataricus*	2V9W	3.00	Ca^{2+}	Ca^{2+}	----	----	A: E106, D7, DCT<br>B.D105, F8, D7, DCT	DCT, primer, template	----
	DNA polymerase IV	*Sulfolobus solfataricus*	2VA2	2.80	----	Ca^{2+}	----	----	B: D105, F8, D7, DCT	DCT, primer, template	----
	DNA polymerase IV	*Sulfolobus solfataricus*	2VA3	2.98	Ca^{2+}	Ca^{2+}	----	----	A: D105, E106, DGT<br>B: D105, F8, D7, DGT	DGT, primer, template	----
	DNA polymerase IV	*Sulfolobus solfataricus*	2XCA	2.50	Mg^{2+}	Mg^{2+}	----	----	A: D105, E106, D7, DGT<br>B: D105, F8, D7, K159,<br>DGT	DGT, primer, (primer DOC),<br>template	----
	DNA polymerase IV	*Sulfolobus solfataricus*	2XCP	2.60	Mg^{2+}	Mg^{2+}	----	----	A: D105, E106, D7, DCP<br>B: D105, F8, D7, DCP	DCP, primer, (modified DOC),<br>template	----
	DNA polymerase IV	*Sulfolobus solfataricus*	3GII	2.60	Ca^{2+}	Ca^{2+}	----	----	A: D105, E106, D7, DGT<br>B: D105, F8, D7, DGT	DGT, primer, (modified 2DA),<br>template	----
	DNA polymerase IV	*Sulfolobus solfataricus*	3GIJ	2.40	Ca^{2+}	Ca^{2+}	----	----	A: E106, D7, K159, DGT<br>B: D105, F8, D7, DGT	DGT, primer, (modified 2DA),<br>template	----
	DNA polymerase IV	*Sulfolobus solfataricus*	3GIK	2.90	Ca^{2+}	Ca^{2+}	----	----	A: E106, D7<br>B: D105, F8, D7, DGT	DGT, primer, (modified DOC),<br>template	----
	DNA polymerase IV	*Sulfolobus solfataricus*	3GIL	2.71	Ca^{2+}	Ca^{2+}	----	----	A: D105, E106, D7,DGT<br>B: D105, F8, D7, DGT	DGT, primer, (modified 2DT),<br>template	----
	DNA polymerase IV	*Sulfolobus solfataricus*	3GIM	2.70	Ca^{2+}	Ca^{2+}	----	----	A: D105, E106, D7,DGT<br>B: D105, F8, D7, DGT	DGT, primer, (modified DDG) ,<br>template	----
	DNA polymerase IV	*Sulfolobus solfataricus*	3KHG	2.96	Ca^{2+}	Ca^{2+}	----	----	A: D105, E106, D7, DGT<br>B: D105, F8, D7, DGT	DGT, primer (modified 2DA),<br>template, AF, EPE	----
	DNA polymerase IV	*Sulfolobus solfataricus*	3KHH	2.70	Ca^{2+}	Ca^{2+}	----	----	A: D105, E106, DGT<br>B: D105, F8, D7, DGT	DGT, primer (modified DOC),<br>template, AF	----
	DNA polymerase IV	*Sulfolobus solfataricus*	3KHL	2.10	Ca^{2+}	Ca^{2+}	----	----	A: D105, E106, D7, TTP<br>B: D105, F8, D7,TTP	TTP, primer (modified DDG),<br>template, AF	----
	DNA polymerase IV	*Sulfolobus solfataricus*	3KHR	2.01	Ca^{2+}	Ca^{2+}	----	----	A: D105, E106, TTP<br>B: D105, F8, D7, TTP	TTP, primer (modified DDG),<br>template, AF	----
	DNA polymerase IV	*Sulfolobus solfataricus*	3M9M	2.90	Ca^{2+}	Ca^{2+}	----	----	A: D105, E106, CTP<br>B: D105, F8, D7, CTP	CTP, primer, template, CPT, GOL	----
	DNA polymerase IV	*Sulfolobus solfataricus*	3M9N	1.93	Ca^{2+}	Ca^{2+}	----	----	A. E106, D7<br>B: D105, F8, D7, CTP	CTP, primer, template, CPT	----
	DNA polymerase IV	*Sulfolobus solfataricus*	3M9O	2.00	Ca^{2+}	Ca^{2+}	----	----	A: E106, D7<br>B: D105, F8, D7, ATP	ATP, primer, template, CPT	----
	DNA polymerase IV	*Sulfolobus solfataricus*	3QZ7	2.00	Ca^{2+}	Ca^{2+}	----	----	A: D105, E106, D7, DCP<br>B: D105, F8, D7, DCP	DCP, primer, template	----
	DNA polymerase IV	*Sulfolobus solfataricus*	3QZ8	2.00	Ca^{2+}	Ca^{2+}	----	----	A: D105, E106, D7, DCP<br>B: D105, F8, D7, DCP	DCP, primer, template	----
	DNA polymerase IV	*Sulfolobus solfataricus*	2R8G	2.70	Ca^{2+}	Ca^{2+}	----	----	A: E106, D7, DGT<br>B: D105, F8, D7, DGT	DGT, primer, template	R332A
	DNA polymerase IV	*Sulfolobus solfataricus*	2R8H	2.48	Ca^{2+}	Ca^{2+}	----	----	A: E106, D7, DGT<br>B: D105, F8, D7, DGT	DGT, primer, template	R332A
	DNA polymerase IV	*Sulfolobus solfataricus*	2R8I	2.38	Ca^{2+}	Ca^{2+}	----	----	A: D105, E106, D7, DTP<br>B: D105, F8, D7, DTP	DTP, primer, template	R332A

	DNA polymerase IV	*Sulfolobus solfataricus*	2UVR	2.90	Ca^{2+}	Ca^{2+}	----	----	A: D105, E106, D7, DGT<br>B: D105, F8, D7, DGT	DGT, primer, template	R332E
	DNA polymerase IV	*Sulfolobus solfataricus*	2UVU	2.70	Ca^{2+}	Ca^{2+}	----	----	A: D105, E106, D7, DGT<br>B: D105, F8, D7, DGT	DGT, primer, template	R332E
	DNA polymerase IV	*Sulfolobus solfataricus*	2UVV	2.20	Ca^{2+}	Ca^{2+}	----	----	A: D105, E106, D7, DGT<br>B: D105, F8, D7, DGT	DGT, primer, template	R332E
	DNA polymerase IV	*Sulfolobus solfataricus*	2UVW	2.09	Ca^{2+}	Ca^{2+}	----	----	A: D105, E106, D7, DGT<br>B: D105, F8, D7, DGT	DGT, primer, template	R332E
	DNA polymerase IV	*Sulfolobus solfataricus*	3PR4	2.65	Ca^{2+}	Ca^{2+}	----	----	A: E106, D7, ATP<br>B: D105, F8, D7, ATP	ATP, primer, template	Y12A
	DNA polymerase IV	*Sulfolobus solfataricus*	3PR5	2.40	----	Ca^{2+}	----	----	B: D105, F8, D7, ATP	ATP, primer, template	Y12A
	DNA polymerase η	*Saccharomycetes cervisiae*	2R8J	3.10	Ca^{2+}	Ca^{2+}	----	----	A: E156, D30, DCP<br>B: D155, M31, D30, DCP	DCP, CPT, primer, template	----
	DNA polymerase η	*Saccharomycetes cervisiae*	2R8K	3.30	Ca^{2+}	Ca^{2+}	----	----	A: D155, E156, D30, DTP<br>B: D155, M31, D30, DTP	DTP, CPT, primer, template	----
	DNA polymerase η	*Saccharomycetes cervisiae*	2WTF	2.50	Ca^{2+}	Ca^{2+}	----	----	A: D155, E156, D30, DTP<br>B: D155, M31, D30, DTP	DTP, primer, template,CPT	----
	DNA polymerase η	*Saccharomycetes cervisiae*	2XGP	2.70	Ca^{2+}	Ca^{2+}	----	----	A: D155<br>B: D155, E156, D30	primer, template	----
	DNA polymerase η	*Saccharomycetes cervisiae*	2XGQ	2.70	Ca^{2+}	Ca^{2+}	----	----	A: D155, E156<br>B: E156, D30	primer, template	----
	DNA polymerase η	*Saccharomycetes cervisiae*	3MFH	2.00	Mg^{2+}	Mg^{2+}	----	----	A: D155, E156, D30, DTP<br>B: D155, M31, D30, DTP	DTP, primer (modified DOC),<br>template, SO_4	K140A, S144W
	DNA polymerase η	*Saccharomycetes cervisiae*	3MFI	1.76	Mg^{2+}	Mg^{2+}	----	----	A: D155, E156, D30, DTP<br>B: D155, M31, D30, DTP	DTP, primer (modified DOC),<br>template, SO_4	K140A, S144W
	DNA polymerase η	*Saccharomycetes cervisiae*	3OHA	2.00	Mg^{2+}	Mg^{2+}	----	----	A; D155, E156, D30, DCP<br>B: D155, M31, D30, DCP	DCP, primer (modified DOC),<br>template, SO_4	K140A, S144W
	DNA polymerase η	*Saccharomycetes cervisiae*	3OHB	2.00	Mg^{2+}	Mg^{2+}	----	----	A: D155, E156, D30, DCP<br>B: D155, M31, D30, DCP	DCP, primer (modified DOC),<br>template, SO_4	K140A, S144W
	DNA polymerase ι	*Homo sapiens*	1T3N	2.30	Mg^{2+}	----	----	----	A: D126, L35, D34, TTP	TTP	----
	DNA polymerase ι	*Homo sapiens*	1ZET	2.30	Mg^{2+}	----	----	----	A: D126, L35, D34	----	----
	DNA polymerase ι	*Homo sapiens*	2ALZ	2.50	Mg^{2+}	Mg^{2+}	----	----	A: D126, E127, D34, DCP<br>B: D126, L35, D34, DCP	DCP, template,primer (modified<br>DOC)	----
	DNA polymerase ι	*Homo sapiens*	2DPI	2.30	Mg^{2+}	Mg^{2+}	----	----	A: D126, E127, D34, TTP<br>B: D126, L35, D34, TTP	TTP, template,primer (modified<br>DOC)	----
	DNA polymerase ι	*Homo sapiens*	2DPJ	2.30	Mg^{2+}	Mg^{2+}	----	----	A: D126, E127, D34, TTP<br>B: D126, L35, D34, TTP	TTP, template,primer (modified<br>DOC)	----
	DNA polymerase ι	*Homo sapiens*	2FLL	2.60	Mg^{2+}	Mg^{2+}	----	----	A: D34, E127, DCP<br>B: D126, L35, D34	DCP, primer (modified DOC),<br>template	----
	DNA polymerase ι	*Homo sapiens*	3G6V	2.20	Mg^{2+}	Mg^{2+}	----	----	A: D34, E127, ATP<br>B: D126, D34, L35, ATP	ATP, primer (modified DOC),<br>template	----
	DNA polymerase ι	*Homo sapiens*	3G6X	2.08	Mg^{2+}	Mg^{2+}	----	----	A: D34, E127, DGT<br>B: D126, D34, L35, DGT	DGT, primer (modified DOC),<br>template	----
	DNA polymerase ι	*Homo sapiens*	3G6Y	2.10	Mg^{2+}	Mg^{2+}	----	----	A: D34, E127, TTP<br>B: D126, L35, D34, TTP	TTP, primer (modifed DOC),<br>template	----
	DNA polymerase ι	*Homo sapiens*	3GV5	2.00	Ca^{2+}	----	----	----	A D34, L35, D126, ADI	ADI, GOL, primer, template	----
	DNA polymerase ι	*Homo sapiens*	3GV7	2.20	Mg^{2+}	Mg^{2+}	----	----	A: D34, TTP<br>B: D34, D126, L35, TTP	TTP, primer, template	----
	DNA polymerase ι	*Homo sapiens*	3GV8	2.00	Mg^{2+}	Mg^{2+}	----	----	A: D126<br>B: D34, D126, L35, DGT	DGT, primer, template	----

	DNA polymerase ι	*Homo sapiens*	3H4D	2.20	Mg^{2+}	Mg^{2+}	----	----	A: D34, E127, D126, DGT<br>B: D34, L35, D126, DGT	DGT, primer (modified DOC), template	----
	DNA polymerase ι	*Homo sapiens*	3MR2	1.83	Mg^{2+}	Mg^{2+}	----	----	A: D115, E116, D13, DZ4<br>B: D115, M14, D13, DZ4	DZ4, primer, template, GOL	----
	DNA polymerase ι	*Homo sapiens*	3MR3	1.75	Mg^{2+}	Mg^{2+}	----	----	A: D115, E116, D13, DZ4<br>B: D115, M14, D13, DZ4	DZ4, primer, template, GOL	----
	DNA polymerase ι	*Homo sapiens*	3MR4	2.15	Mg^{2+}	Mg^{2+}	----	----	A: D115, E116, D13, DZ4<br>B: D115, M14, D13, DZ4	DZ4, primer, template, 3D1, DZ4, EDO, Co^{2+}, DTT, GOL	----
	DNA polymerase ι	*Homo sapiens*	3MR5	1.80	Mg^{2+}	Mg^{2+}	----	----	A: D115, E116, D13, XG4<br>B: D115, M14, D13, XG4	XG4, primer, template, GOL	----
	DNA polymerase ι	*Homo sapiens*	3OSN	1.90	Mg^{2+}	Mg^{2+}	----	----	A: E127, D34, TTP<br>B: D126, D34, L35, TTP	Na^{+}, TTP	----
	DNA polymerase ι	*Homo sapiens*	3Q8P	1.95	Mg^{2+}	----	----	----	B: D126, L35, D34, DCP	DCP, primer, template	----
	DNA polymerase ι	*Homo sapiens*	3Q8Q	2.03	Mg^{2+}	----	----	----	B: L35, D126, DTP	DTP, primer, template	----
	DNA polymerase ι	*Homo sapiens*	3Q8R	2.45	Mg^{2+}	----	----	----	B: L35, D34, DGT	DGT, primer, template	----
	DNA polymerase ι	*Homo sapiens*	3Q8S	2.09	Mg^{2+}	----	----	----	A: D126, L35, D34, TTP	TTP	----
	DNA polymerase ι	*Homo sapiens*	3MR6	1.90	Mg^{2+}	Mg^{2+}	----	----	A: D115, E116, D13, XG4<br>B: D115, M14, D13, XG4	XG4, primer, template, GOL	C406M
	DNA polymerase Rev1	*Saccharomycetes cervisiae*	3OSP	2.50	Mg^{2+}	Mg^{2+}	----	----	A: D362, D467, E468, DCP<br>B: D362, F363, D467, DCP	DCP, template, primer (modified DOC)	----
	DNA polymerase Rev1	*Saccharomycetes cervisiae*	2AQ4	2.32	Mg^{2+}	Mg^{2+}	----	----	A: D467, E468, D362, DCP<br>B: D362, F363, D467, DCP	DCP	----
	DNA polymerase Rev1	*Saccharomycetes cervisiae*	3BJY	2.41	Mg^{2+}	Mg^{2+}	----	----	A: D362, D467, E468, DCP<br>B: D362, F363, D467, DCP	DCP, template, primer (modified DOC)	D20N, D212N, I539F, Q540E, V541A, V542E
DNA-dependent RNA polymerases	DNA-dependent RNA polymerase	enterobacteria phage T7	1S76	2.88	Mg^{2+}	Mg^{2+}	----	----	A: D812, D537, APC<br>B: D812, G538, D537, APC	APC	----
	DNA-dependent RNA polymerase	enterobacteria phage T7	1S77	2.69	Mg^{2+}	----	----	----	B: F812, G583, D537, POP	POP, 3 polyribonucleotides	----
	DNA-dependent RNA polymerase	enterobacteria phage N4	3Q22	2.11	----	Mg^{2+}	----	----	B: D951, G560, D559, GTP	GTP, polydeoxyribonucleotides, 2HP	D222A, D327, Y567A
	DNA-dependent RNA polymerase	enterobacteria phage N4	3Q23	1.80	Mn^{2+}	Mn^{2+}	----	----	A: D951, D559, G2P<br>B: D951, G560, D559, G2P	G2P, polyribonucleotides	D222A, D327A, S565G, Y567A

[1] (?) indicates that the ion is a catalytic ion but it is not in the catalytic site.

[2]Abbreviations for the ligands

914	[(2r,5r)-5-(6-aminopurin-9-yl)-4-fluoro-2,5-dihydrofuran-2-yl]oxymethyl-[hydroxy(phosphonooxy)phosphoryl]oxy-phosphinic acid
1PE	pentaethylene glycol
23T	2',3'-dideoxy-thymidine-5'-triphosphate
2DA	2',3'-dideoxyadenosine-5'-monophosphate
2DT	3'-deoxythymidine-5'-monophosphate
2HP	dihydrogenphosphate ion
3D1	(2r,3s,5r)-5-(6-amino-9h-purin-9-yl)-tetrahydro- 2-(hydroxymethyl)furan-3-ol
4DG	2-[(2-amino-6-oxo-1,6-dihydro-9h-purin-9-yl)methoxy]ethyl dihydrogen phosphate
4DG	2-[(2-amino-6-oxo-1,6-dihydro-9h-purin-9-yl)methoxy]ethyl dihydrogen phosphate
8OG	8-oxo-2'-deoxy-guanosine-5'-monophosphate
ACT	acetate ion
ADI	2',3'-dideoxyadenosine-5'-diphosphate
AF	2-aminofluorene
APC	diphosphomethylphosphonic acid adenosyl ester
ATM	3'-azido-3'-deoxythymidine-5'-monophosphate
ATP	adenosine-5'-triphosphate
BAP	1,2,3-trihydroxy-1,2,3,4-tetrahydrobenzo[a]pyrene

Ca^{2+}	calsium ion
Co^{2+}	cobalt ion
CPT	cisplatin
CTP	cytidine-5'-triphosphate
D3T	2',3'-dideoxy-thymidine-5'-triphosphate
DAD	2',3'-dideoxyadenosine-5'-triphosphate
DCP	2'-deoxycytidine-5'-triphosphate
DCT	2',3'-dideoxycytidine 5'-triphosphate
DDG	2',3'-dideoxy-guanosine-5'-monophosphate
DDY	2',3'-dideoxycytosine-5'-diphosphate
DG	2'-deoxyguanosine-5'-monophosphate
DG3	2'-3'-dideoxyguanosine-5'-triphosphate
DGT	2'-deoxyguanosine-5'-triphosphate
DOC	2',3'-dideoxycytidine-5'-monophosphate
DTP	2'-deoxyadenosine 5'-triphosphate
DTT	2,3-dihydroxy-1,4-dithiobutane
DZ4	2'-deoxy-5'-o-[(r)-hydroxy{[(r)-hydroxy(phosphonooxy)phosphoryl]amino}phosphoryl]adenosine
EDO	1,2-ethanediol
EPE	4-(2-hydroxyethyl)-1-piperazine ethanesulfonic acid
GOL	glycerol
HXB	4'-methylthymidine 5'-(tetrahydrogen triphosphate)
HXZ	4'-ethylthymidine 5'-(tetrahydrogen triphosphate)
MES	2-(n-morpholino)-ethanesulfonic acid
Mg^{2+}	magnesium ion
Mn^{2+}	manganese ion
MRG	n2-(3-mercaptopropyl)-2'-deoxyguanosine-5'-monophosphate
PEG	di(hydroxyethyl)ether
PGE	triethylene glycol
POP	pyrophosphate 2-
PPF	phosphonoformic acid
SO4	sulfate ion
SSJ	2'-deoxy-5-[(1-hydroxy-2,2,5,5-tetramethyl-2,5-dihydro-1h-pyrrol-3-yl)ethynyl]uridine 5'-(tetrahydrogen triphosphate)
SUC	sucrose
SWE	alpha-d-fructofuranosyl alpha-d-glucopyranoside
TMP	thymidine-5'-phosphate
TTP	thymidine-5'-triphosphate
XG4	2'-deoxy-5'-o-[(r)-hydroxy{[(r)-hydroxy(phosphonooxy)phosphoryl]amino}phosphoryl]guanosine
XJS	2'-deoxy-5-[9-(3-{[4-(diethylamino)-4-oxobutanoyl]amino}propyl)-18-ethyl-5,8,14,17-tetraoxo-4,9,13,18-tetraazaicos-1-yn-1-yl]uridine 5'-(tetrahydrogen triphosphate)
ZP4	[[[[(2r,3s,4r,5r)-5-(6-aminopurin-9-yl)-3,4-dihydroxy-oxolan-2-yl]methoxy-hydroxy-phosphoryl]oxy-hydroxy-phosphoryl]oxy-hydroxy-phosphoryl] [(2s,3s,5r)-3-azido-5-(5-methyl-2,4-dioxo-pyrimidin-1-yl)oxolan-2-yl]methyl hydrogen phosphate
